# Cancer-associated fibroblasts influence Wnt/PCP signaling in gastric cancer cells by cytoneme-based dissemination of ROR2

**DOI:** 10.1073/pnas.2217612120

**Published:** 2023-09-18

**Authors:** Sally Rogers, Chengting Zhang, Vasilis Anagnostidis, Corin Liddle, Melissa L. Fishel, Fabrice Gielen, Steffen Scholpp

**Affiliations:** ^a^Living Systems Institute, University of Exeter, Exeter EX4 4QD, United Kingdom; ^b^Bioimaging Centre, University of Exeter, Exeter EX4 4QD, United Kingdom; ^c^Indiana University, School of Medicine, Indianapolis, IN 46202-308

**Keywords:** Wnt/PCP signalling, cytoneme, gastric cancer, cell migration, morphogen

## Abstract

Cancer-associated fibroblasts (CAFs) are a crucial component in the tumor microenvironment influencing cancer progression. Besides shaping the extracellular matrix, these fibroblasts provide signaling factors to facilitate tumor survival and alter tumor behavior. In gastric cancer, one crucial signaling pathway influencing invasion and metastasis is the Wnt/Planar Cell Polarity (PCP) signaling. The crucial PCP ligand in this context is WNT5A, which is produced by the CAFs, and gastric cancer cells react upon this signal by enhanced polarized migration. Why gastric cancer cells respond to this signal is still unclear, as their expression level for the central WNT5A receptor, ROR2, is very low. Here, we show that CAFs display long and branched filopodia that form an extensive, complex network engulfing gastric cancer cells, such as the gastric cancer cell line AGS. CAFs have a significantly higher expression level of ROR2 than normal gastric fibroblasts and AGS cells. By high-resolution imaging, we observe a direct transfer of fluorescently tagged ROR2 from CAF to AGS cells by signaling filopodia, known as cytonemes. Surprisingly, we find that the transferred ROR2 complexes can activate Wnt/JNK signaling in AGS cells. Consistently, blockage of ROR2 function in the CAFs leads to reduced paracrine Wnt/JNK signaling, cell polarization, and migration of the receiving AGS cells. Complementary, enhanced migration via paracrine ROR2 transfer was observed in a zebrafish in vivo model. These findings demonstrate a fresh role for cytoneme-mediated signaling in the tumor microenvironment. Cytonemes convey Wnt receptors from CAFs to gastric cancer cells, allowing them to respond to Wnt/PCP signals.

## Significance

Fibroblasts are the prevalent cells in the stroma of gastric carcinoma. These specialized fibroblasts provide signaling factors influencing tumor behavior. Here, we show that these cancer-associated fibroblasts provide both the signaling protein WNT5A and its receptor ROR2 to gastric cancer. We further demonstrate that the transferred receptor ROR2 remains active in the tumor cells to influence cell polarization and migration. Our findings suggest a fresh mechanistic understanding of how signaling components are conveyed in a tumor; thus, our findings add an additional role of the cancer stroma in influencing invasion and metastasis.

The Wnt signaling network plays a crucial role in orchestrating the development and progression of many cancers (1). Within this network, the Wnt/Planar Cell Polarity (PCP) signaling pathway has been implicated in tumor cell migration and invasion (2). To activate the Wnt/PCP pathway, Wnt ligands such as WNT5A are considered to bind to the receptor tyrosine kinase–like orphan receptor 2 (ROR2) or RYK in concert with Frizzled receptors (FZD) (3, 4). Subsequently, WNT5A/ROR2 interaction leads to the activation of c-Jun N-terminal kinase (JNK) signaling, one effector associated with cell polarity, cytoskeleton rearrangements, and cell migration (5, 6). WNT5A expression is associated with several cancers, resulting in constitutive activation of ROR2-mediated signaling and contributing to tumor progression (7–9). In gastric cancer, WNT5A is highly expressed in the stroma (10), contributes to the tumor microenvironment, and is explicitly up-regulated in cancer-associated fibroblasts (CAFs) compared to fibroblasts isolated from the normal gastric stroma (11). However, how gastric cancer cells can respond to WNT5A signaling remains unclear, given that the main receptor, ROR2, is commonly down-regulated in these cells (12, 13).

CAFs display an increased number of filopodia, which can influence the behavior of the tumor cells. For example, filopodia have been shown to increase metastasis of several different cancer cell types (14). Furthermore, actin filament bundling proteins like Fascin are often overexpressed in cancers, which increases the length and number of filopodia. This results in the increased ability of cells to migrate and invade, which generally correlates with poor survival prognosis (15, 16). To date, the molecular function of the filopodial network generated by CAFs is poorly understood. Recently, actin-containing filopodia, also known as cytonemes, have been shown to facilitate the transport of signaling components in development and diseases (17). Specifically, Wnt3 spreading in gastric cancer is promoted by Flot2-positive cytonemes. The number and length of Wnt3 cytonemes correlate with gastric cancer cell survival and proliferation (18). It is unclear whether CAFs can form cytonemes to distribute signaling components in the tumor microenvironment.

Here, we show that ROR2 is up-regulated on pancreatic and gastric CAFs compared to the gastric cancer cell lines and normal gastric fibroblasts. We find that ROR2 is localized on the plasma membrane and on long filopodia of CAFs, which form a dense filopodia network interacting with the cancer cells. Surprisingly, we observe that ROR2 is transported from CAFs to gastric cancer cells via this network. High-resolution microscopy revealed that filopodia tips from ROR2-overexpressed cells bud off and fuse with the plasma membrane of the receiving gastric cancer cells. To further elucidate the hand-over of the endogenous receptor, we used a pH-dependent antibody against ROR2 and found that the WNT5-interacting cysteine-rich domain (CRD) remains in the extracellular space, whereas the kinase domain of ROR2 is orientated to the receiving cell cytoplasm allowing signaling into the receiving cell. After endocytosis of the active receptor, the intracellular kinase domain remains in the cytoplasm to allow prolonged signaling. Indeed, when we used a reporter system to monitor the activation of the Wnt/JNK signaling pathway, we found that PCP signal activation is proportional to the amount of delivered ROR2. By using an advanced biochip and a hydrogel-based three-dimensional (3D) cell culture approach, we further provide evidence that CAF-produced cytonemes can induce cell polarization and directional migration in a ROR2-dependent manner in both in vitro and zebrafish embryos.

Taken together, our results provide evidence of an intercellular transport mechanism for membrane-spanning proteins in the tumor microenvironment: The cognate WNT5A coreceptor ROR2 can be transported from CAFs to receiving tumor cells on signaling filopodia. The cytoneme-delivered ROR2 activates the Wnt/PCP pathway in receiving cells. The spreading of the receptor can explain how gastric cancer cells, low in ROR2, can respond to a WNT5A-high microenvironment and change into an invasive phenotype.

## Results

### CAFs Display a Filopodia Network and Express a High Level of WNT5A and ROR2.

**Fig. 1.**
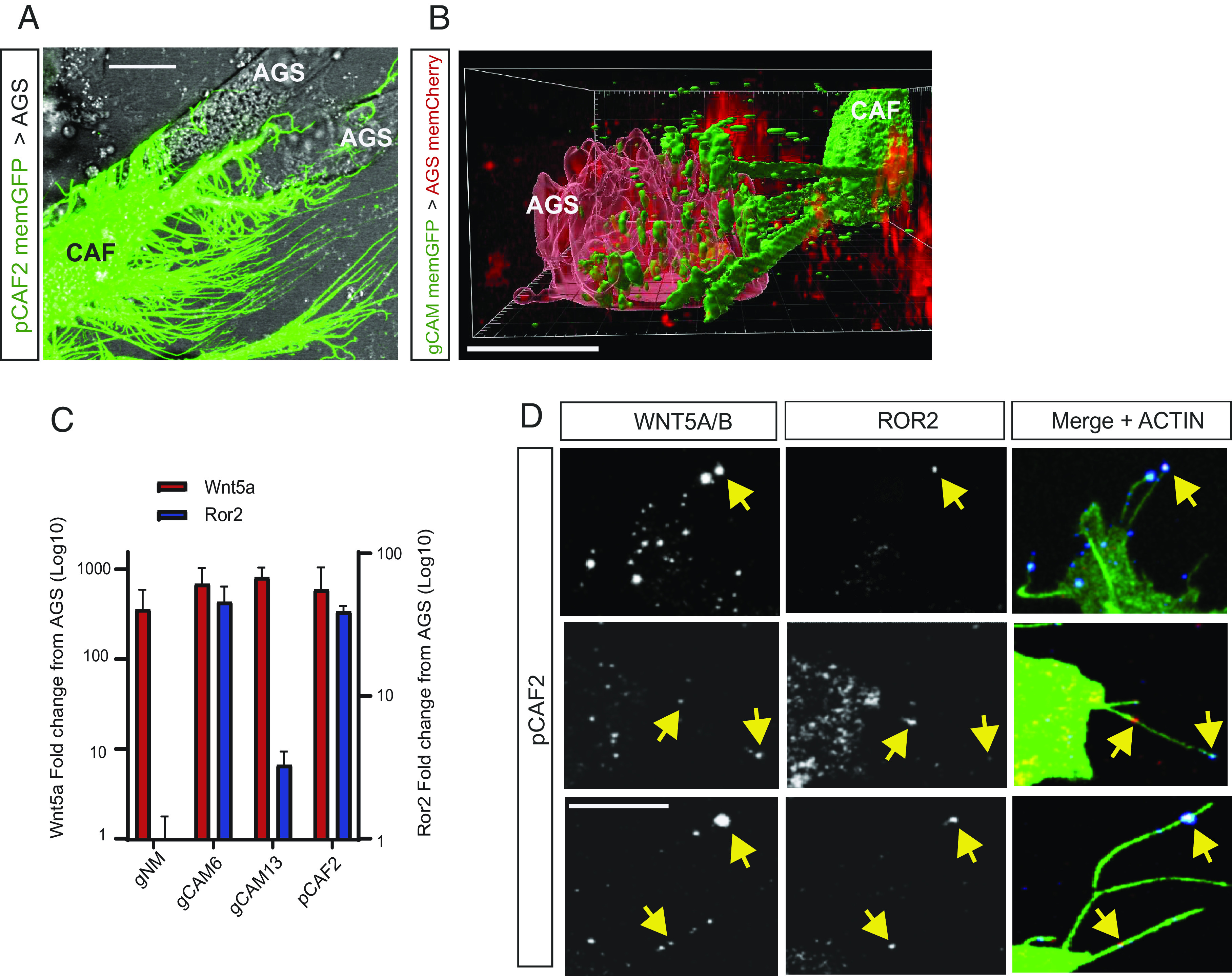
CAFs form extensive filopodial contacts with AGS cells and have up-regulated ROR2 expression on cytoneme tips. (*A*) pCAF cells (C) transfected with membrane GFP form an extensive and complex network of filopodia which engulf neighboring AGS cells (A) in 2D cell culture. The scale bar represents 10 µm. (*B*) primary gastric cancer–associated myofibroblasts (gCAM) (C) transfected with membrane GFP extend multiple filopodia in 3D culture that contact AGS cells (A) transfected with membrane mCherry, and transport multiple membrane-bound vesicles from producing to receiving cell (*SI Appendix*, Fig. S1*A*). The scale bar represents 10 µm. (*C*) mRNA levels of the indicated gene are shown as fold change away from AGS. Wnt5a is shown in red, with values on the left *Y* axis. ROR2 is shown in blue, with values on the right *Y* axis. gNM, primary gastric normal myofibroblasts; gCAM6, primary gastric cancer–associated myofibroblast patient 6; gCAM13, primary gastric cancer–associated myofibroblast patient 13; pCAF2, pancreatic CAF cell line 2. Values shown were calculated using the 2^−ΔΔCt^ method and are plotted on a log10 scale. Comparison of 40-Ct values for all cells is shown in *SI Appendix*, Fig. S1 *B* and *C*. (*D*) Immunofluorescence on pCAF2 cells fixed using a previously described method to preserve filopodia. The left-hand column indicates WNT5A/B antibody staining, the center panel indicates ROR2 antibody staining, and the right-hand panel indicates WNT5A/B and ROR2 antibody staining (blue and red, respectively) merged with Phalloidin FITC shown in green. Three representative experiments are shown, and yellow arrowheads indicate WNT5A/ROR2 clusters. Antibody controls are shown in *SI Appendix*, Fig. S1 *G* and *H*. The scale bar represents 10 µm.

We have previously shown that actin-based signaling filopodia, also known as cytonemes, can mediate paracrine Wnt signaling in both development and gastric cancer (18–20). To determine the extent of filopodial contacts between CAFs and gastric cancer cells, we transfected pCAF2 cells with membrane GFP and cocultured them with untransfected gastric cancer cell line AGS. pCAF2 cells form extensive, numerous, and complex filopodial networks with AGS cells in 2D culture, with evidence of engulfment and wrapping of the AGS cells (Fig. 1*A*). In addition, numerous vesicles containing CAF cell membrane can be identified in receiving AGS cells that have been contacted by fibroblast filopodia in 3D hydrogel-based coculture (Fig. 1*B* and Movie S1 and *SI Appendix*, Fig. S1*A*).

Next, we addressed the question of whether this filopodial network can be used to disseminate Wnt signaling components. Therefore, we first analyzed the expression of a crucial PCP ligand, WNT5A. We find that WNT5A expression is higher in gastric CAFs than in the gastric tumor cell line AGS and surrounding normal fibroblasts, which has been suggested previously (11). *WNT5A* mRNA was shown to be 357-fold higher in primary gastric normal myofibroblasts (gNM) compared to AGS cells and over 800-fold higher in primary gastric cancer–associated myofibroblasts (gCAM) and a pancreatic cancer–associated fibroblast cell line (pCAF2) compared to AGS cells using qRT-PCR (Fig. 1*C* and *SI Appendix*, Fig. S1*B*). A flow cytometry–based analysis validated this finding at the protein level (*SI Appendix*, Fig. S1*D*). Next, we analyzed the expression pattern of the WNT5A coreceptor ROR2 (3). By using qRT-PCR, we found that *ROR2* mRNA expression is significantly increased in gCAM and pCAF2 compared to both AGS and gNM (Fig. 1*C* and *SI Appendix*, Fig. S1*C*). Furthermore, the expression of *ROR2* mRNA varied between gCAM isolated from different patients but was higher overall in CAFs than gNM, regardless of origin. We confirmed that mRNA expression correlated with protein expression using western blotting (*SI Appendix*, Fig. S1 *E* and *F*). Our results are supported by previous reports indicating that the ROR2 is down-regulated in gastric cancer cells (12, 13). Therefore, the primary aim of this study was to investigate how gastric cancer cells respond to a WNT5A-high environment despite lacking one of the most important WNT5A receptors.

### ROR2/WNT5A Complexes Are Transferred from CAF to AGS Cells via Cytonemes.

Our previous studies have identified several Wnt signaling components localized to signaling filopodia, also known as cytonemes in cancer cells (18, 20). We, therefore, established an IF method that fixes these filopodia to examine the cellular localization of Wnt components (21). We applied that protocol to address the question of whether endogenous ROR2 and WNT5A are transported on cytonemes. We found WNT5A along the length of pCAF2 filopodia, including at the tips of these actin-based structures (Fig. 1*D*). To our surprise, we further observed that these WNT5A clusters colocalize with ROR2 (Fig. 1*D* and *SI Appendix*, Fig. S1 *F* and *G*).

**Fig. 2.**
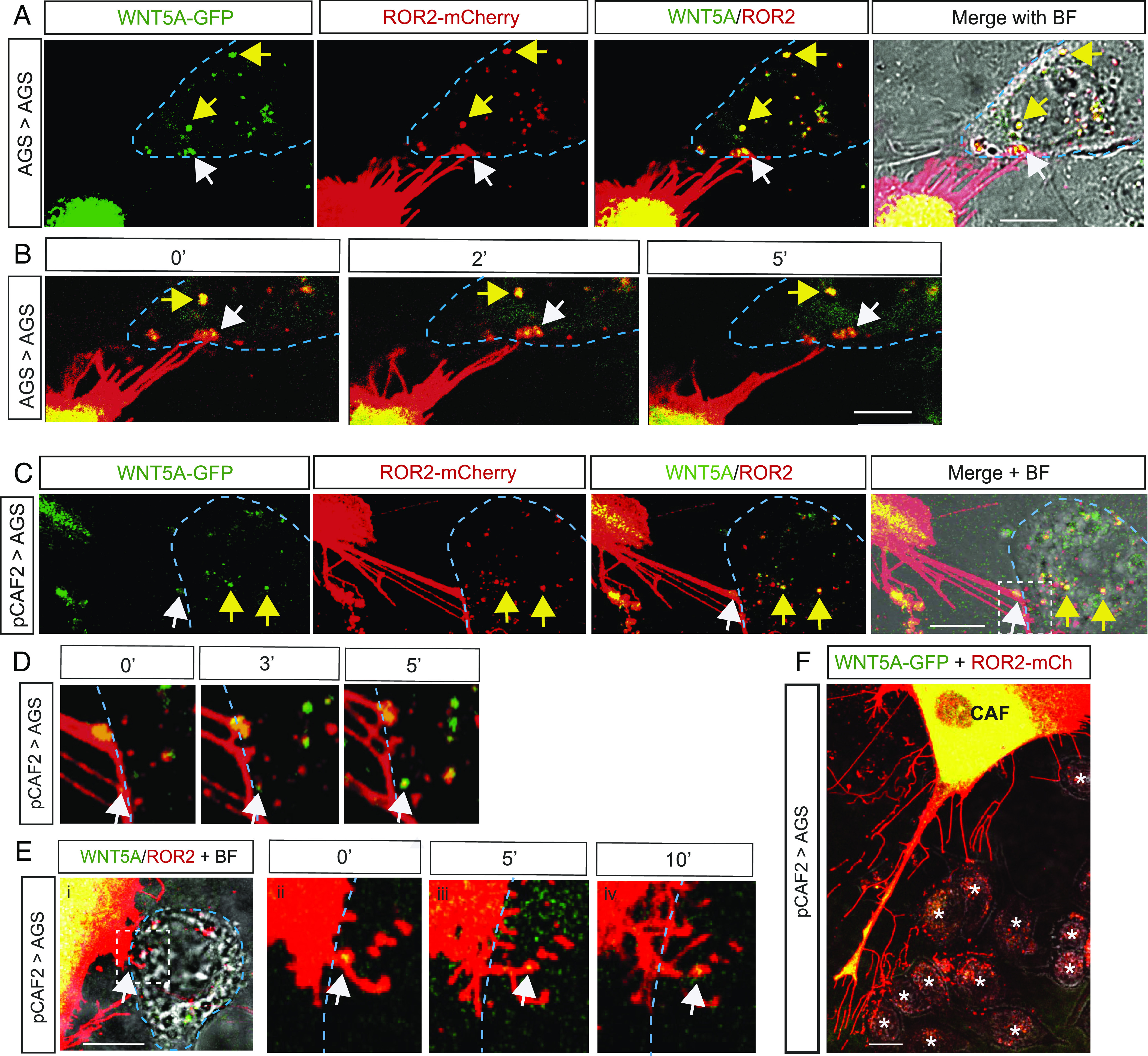
ROR2 and WNT5A complexes are transported from producing pCAF2 cells to receiving AGS cells. (*A*) AGS cell expresses ROR2-mCherry and WNT5A-GFP on cytoneme tips (white arrowhead), which can be seen contacting a neighboring AGS receiving cell identified in the BF image and indicated by the blue dashed line. Other ROR2/WNT5A complexes from the producing cell can be seen localizing in the receiving cell, as indicated by the yellow arrows. The scale bar represents 10 µm. (*B*) Time-lapse images, as indicated in minutes, show a ROR2/WNT5A complex (white arrowhead) leaving the cytoneme and colocalizing in the receiving cell shown in *A*. Other ROR2/WNT5A complexes from the producing cell can be seen continuing to colocalize in the receiving cell over time, as indicated by the yellow arrow. The scale bar represents 10 µm. (*C*) pCAF2 cell expresses ROR2-mCherry and WNT5A-GFP on cytoneme tips (white arrowhead), which can be seen contacting a neighboring AGS receiving cell identified in the BF image and indicated by the blue dashed line. Other ROR2/WNT5A complexes from the producing cell can be seen localizing in the receiving cell, as indicated by the yellow arrows. The scale bar represents 10 µm. (*D*) Time-lapse images, as indicated in minutes, of the area denoted by the white dashed box in *C*. A ROR2/WNT5A complex (white arrowhead) leaves the cytoneme and colocalizes in the receiving cell shown in *C*. (*E*) A further example of the transport of ROR2/WNT5A complexes from pCAF2-producing cells to AGS-receiving cells. Images as described for *C* and *D*. (*E*, *ii*–*iv*) show the time-lapse images of the white boxed area in *E*, *i*. The scale bar represents 10 µm. (*F*) pCAF2 cell (C) transfected with ROR2 mCherry and WNT5A GFP transport complexes to receiving AGS cells (A) over a large distance. The scale bar represents 10 µm.

To determine whether ROR2 and WNT5A could be transported to a receiving cell, fluorescently labeled ROR2 and WNT5A were initially coexpressed in AGS cells and cocultured with nontransfected AGS cells. Receptor–ligand complexes were observed to be not only colocalizing along cytonemes but also colocalizing in the neighboring AGS cells (Fig. 2*A* and *SI Appendix*, Fig. S2 *A* and *B*). We observed the same phenomena occurring between pCAF2 cells overexpressing WNT5A and ROR2 (Fig. 2*C*). Time-lapse images revealed that ROR2/WNT5A complexes can be directly transferred via both AGS and pCAF2- producing cell cytonemes to a receiving AGS cell (Fig. 2 *B*, *D*, and *E*). Furthermore, we found stable receptor–ligand complexes in receiving cells for over 30 min before phototoxicity and photobleaching prevent further measurement. We hypothesized that in this way, the ROR2-low cells, like the gastric tumor cells AGS, could increase their responsiveness to fibroblast-produced WNT5A as they are receiving a functional receptor. In addition, we show that pCAF2 filopodia transport ROR2/WNT5A complexes over considerable distances, extending in excess of 100 μm to several receiving cells (Fig. 2*F*). We found that WNT6 can also be transported between AGS cells via cytonemes (*SI Appendix*, Fig. S2*G*). However, in contrast to WNT5A, WNT6 does not colocalize with ROR2 in the receiving cell as determined by the Pearson correlation coefficient (*SI Appendix*, Fig. S2*H*). This suggests that this transport mechanism is specific between ROR2 and WNT5A.

Posttranslational modification of Wnt proteins by porcupine-mediated palmitoleoylation is essential for Wnt secretion (18). Therefore, we treated AGS cells transfected with ROR2 and WNT5A with the porcupine inhibitor IWP2 to block the transport of WNT5A to the receiving cell. Interestingly, labeled ROR2 was still observed in the receiving cell, indicating that ROR2 can be shuttled to the cell surface and transported to a neighboring cell in the presence of non-palmitoleated WNT ligands (*SI Appendix*, Fig. S2*E*).

### ROR2/WNT5A Complexes Are Endocytosed with Producing Cell Membrane Using a Dynamin2-Dependent Mechanism in the Receiving Cells.

**Fig. 3.**
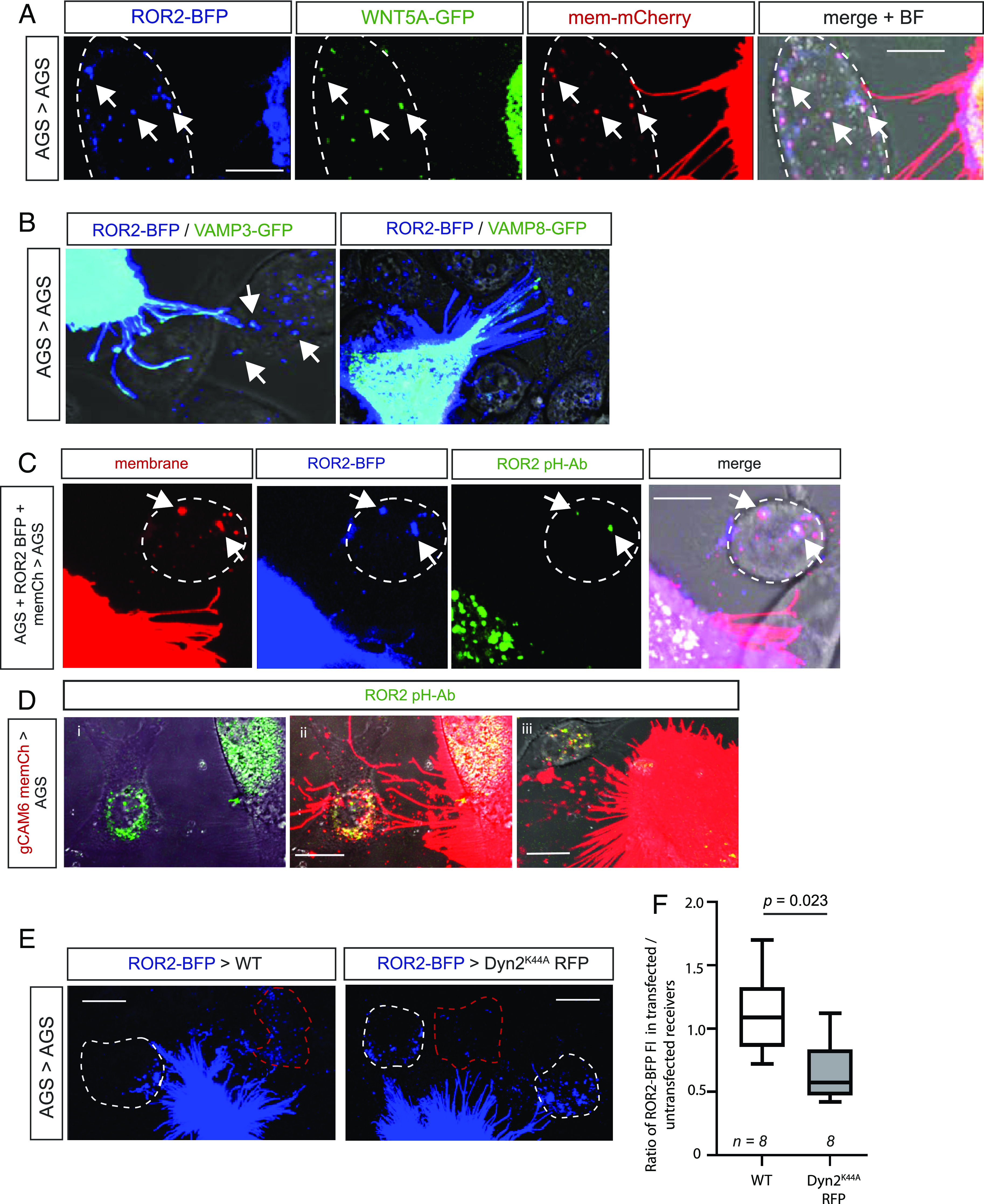
WNT5A/ROR2 complexes are endocytosed by receiving cells and exist in a conformation that enables signaling. (*A*) Confocal image of an AGS cell transiently transfected with ROR2-BFP, WNT5A-GFP, and membrane mCherry. ROR2/WNT5A/membrane marker complexes in a receiving, untransfected AGS cell, as determined by the bright-field (BF) image, are indicated by white arrowheads. The scale bar represents 10 µm. An orthogonal view is shown in *SI Appendix*, Fig. S3*A*. (*B*) AGS cells were transiently transfected with ROR2-BFP and either VAMP3-GFP or VAMP8-GFP and live imaged using confocal microscopy. The scale bar represents 10 µm (*C*) AGS cells transfected with membrane mCherry and ROR2-BFP were cocultured with untransfected AGS cells, incubated in anti-ROR2 antibody labeled with a pH-dependent GFP dye for 20 h, and live imaged by confocal microscopy. The scale bar represents 10 µm. (*D*) gCAM6 cells transfected with membrane mCherry were cocultured with AGS cells, incubated in anti-Ror2 antibody labeled with a pH-dependent GFP dye for 20 h, and live imaged by confocal microscopy. *i*) and *ii*) shows the same frame, *iii*) shows a second example. The scale bar represents 10 µm. (*E*) AGS cells were transiently transfected with ROR2-BFP and cocultured with AGS cells expressing either membrane mCherry or RFP Dyn2^K44A^ for 24 h. Cells were live imaged using confocal microscopy. The amount of ROR2-BFP in transfected AGS (red dashed line) was quantified. Full images are shown in *SI Appendix*, Fig. S3*D*. The scale bar represents 10 µm. (*F*) The ratio of the amount of ROR2 in the transfected receiving cell compared to the untransfected receiving cell was calculated for control (membrane mCherry) and Dyn2^K44A^ cells. Significance was determined using an unpaired Student *t* test.

To further investigate the mechanism of transport from producing to the receiving cell, we cotransfected AGS cells with ROR2 tagged with blue fluorescent protein (ROR2-BFP), WNT5A-GFP, and membrane-tethered GPI-mCherry and cocultured these triple transfected cells with untransfected AGS cells. We found that all three fluorescently tagged proteins are transported via cytonemes from the producers to the receivers (Fig. 3*A*). We further observed clusters of ROR2-BFP/WNT5A-GFP in the receiving cells (white arrowheads). As these clusters also colocalize with our mCherry-positive membrane marker, we conclude that these are internalized vesicles containing predominantly membrane from the producing cells. This observation, together with the results above, suggests that part of the cytoneme tip buds off, is handed over, and, subsequently, internalized by the receiving cell (*SI Appendix*, Fig. S3*A*). In Drosophila, the SNARE protein snyaptobrevin, a vesicle-associated membrane protein (VAMP), facilitates Hh cytoneme contacts in the wing imaginal disc (22). In accordance with these findings, we find the exocytosis proteins VAMP3 and VAMP8 localizing to the producing AGS cell cytoneme tips (Fig. 3*B*). In addition, VAMP3 (but not VAMP8) was found to colocalize with ROR2 in the receiving cell (white arrows).

Having found that ROR2-positive cytoneme vesicles are internalized by the receiving cell, we next asked how a membrane-spanning receptor is presented in the membrane during the transport. Therefore, we coupled an antibody against the extracellular domain of ROR2 to a pH-sensitive fluorescent conjugate. In detail, an anti-ROR2-CRD antibody and a control antibody were labeled with a pH-sensitive pHrodo™ Green STP ester dye, which fluoresces at acidic pH. Specificity of the labeled antibody (ROR2-pH-Ab) for endocytosed (but not surface) ROR2 was confirmed in both AGS and CAF cells (*SI Appendix*, Fig. S3 *B* and *C*). First, we transfected AGS cells with membrane mCherry and ROR2-BFP and incubated these cells with ROR2-pH-Ab. After 20 h, we imaged live cells by confocal microscopy. As previously shown, we found that the transfected cells transfer ROR2-BFP together with mCherry-labeled membrane to the receiving cell. In the receiving cells, we also observed a signal from the ROR2-pH-Ab colocalizing with the ROR2-BFP (Fig. 3*C* and *SI Appendix*, Fig. S3*C*), indicating that the ROR2-pH-Ab plus ROR2-BFP is taken up by endocytosis and routed to an acidic environment of for example, a late endosome or lysosome. Most importantly, the ROR2-pH-Ab used binds to the CRD domain of ROR2, and therefore, it is this portion of the receptor that is orientated into the acidic lumen of the endosome. This conformation results in the signaling-active kinase domain of the ROR2 being positioned in the cytoplasm of the receiving cell and, therefore, allows activation of ROR2 downstream signaling in the receiving cell.

To test whether endogenous ROR2 is handed over similarly, we next cocultured primary gastric CAM with AGS cells and incubated with ROR2-pH-Ab (Fig. 3*D*). In the control experiments, no staining was detected in the coculture incubated with the control pH antibody (*SI Appendix*, Fig. S3*B*) or in wild-type AGS cells (*SI Appendix*, Fig. S3*C*). However, we find that endogenous levels of ROR2 produced by primary gCAM could be detected colocalizing with our pH-dependent ROR2 antibody in the ROR2-producing CAMs (Fig. 3*D*). Strikingly, we also find a signal of the ROR2-pH-Ab together with producing cell membrane in AGS receiving cells. Based on these findings, we propose that ROR2-positive vesicles are taken up by endocytosis into the receiving cell. To test this hypothesis, we next blocked Dynamin-dependent endocytosis in the receiving cell to reduce the uptake of transferred ROR2. We find that receiving cells transfected with a dominant negative form of Dynamin 2 (Dyn2^K44A^) take up significantly less ROR2 than cells transfected with membrane mCherry as a control (Fig. 3 *E* and *F* and *SI Appendix*, Fig. S3*D*).

Taken together, these results have led us to propose an unexpected intercellular transport mechanism for signaling receptors (*SI Appendix*, Fig. S3*E*). ROR2 is loaded on cytonemes of the producing cell and then transported to a receiving cell (1). At contact, the ROR2-positive tip of the cytoneme, including the membrane, buds off and fuses with the receiving cell, presumably due to SNARE protein function (2). Dynamin2-mediated endocytosis follows, resulting in an uptake of ROR2 complexes into receiving cells (3). During this transport, the kinase domain orientated toward the cell cytoplasm of the producing cell, and after the fusion, toward the cytoplasm of the receiving cell. Complementary, the CRD domain faces the extracellular lumen and, after endocytosis, resides within the signaling endosome (4). We hypothesize that activated ROR2 can signal to the producing cell and, after hand-over, to the receiving cell.

### CAF-Produced ROR2 Can Induce JNK Signaling in Recipient AGS Cells.

**Fig. 4.**
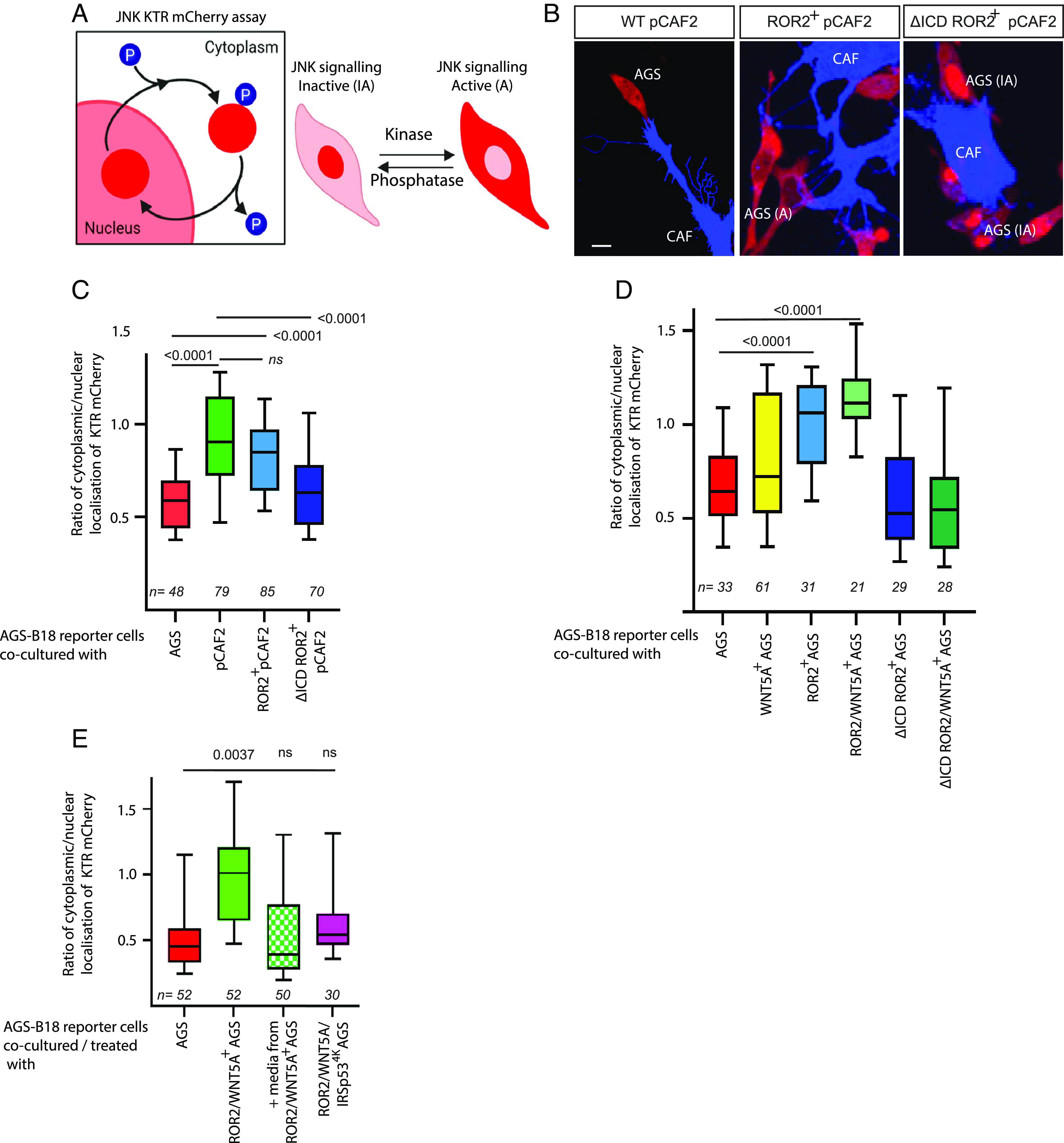
ROR2 induces JNK signaling in receiving AGS cells. (*A*) Schematic depicting the JNK signaling assay (adapted from ref. 25). Upon activation of the JNK signaling pathway, the mCherry reporter protein translocates from the nucleus to the cytoplasm of the cell. (*B*) Representative confocal images of AGS-B18 cells cocultured with wild-type pCAF2 transfected with either membrane GFP (*Left*), ROR2 BFP (*Center*), or dCD ROR2 BFP (*Right*). The scale bar represents 10 μm. (*C*) Quantification of the cytoplasmic to nuclear ratio of the JNK reporter signal in receiving AGS-B18 reporter cells cocultured with pCAF2 cells as described in (*B*). Reporter cells were cocultured with wild-type (WT) AGS cells as a control. Boxes represent 95% quartile, the centerline indicates the mean, and the whiskers indicate the range. n = number of receiving cells quantified. Results are from three independent experiments. Significance was calculated using an unpaired *t* test between two conditions. (*D*) Quantification of the cytoplasmic to the nuclear ratio of JNK reporter signal in AGS-B18 reporter cells cocultured with AGS cells transfected with either membrane GFP (WT AGS), ROR2, ROR2, and WNT5A, ROR2 lacking the cytoplasmic domain (dCD ROR2) or dCD ROR2plus WNT5A as indicated. n = number of receiving cells quantified. Results are from three independent experiments. Significance was calculated using an unpaired *t* test between two conditions. (*E*) Quantification of the cytoplasmic to nuclear ratio of JNK reporter signal in AGS-B18 reporter cells cocultured with AGS cells transfected with either membrane GFP (WT AGS), ROR2 and WNT5A, or ROR2, WNT5A, and dnIRSp53^4K^ to block cytoneme formation as indicated. AGS-B18 reporter cells were also cultured in media only taken from AGS cells transfected with ROR2 and WNT5A for 24 h as indicated by MEDIA. n = number of receiving cells quantified. Significance was calculated using an unpaired *t* test between each two conditions.

After establishing the mechanism of how ROR2 can be transferred to neighboring cells in the tumor microenvironment, we asked the question of whether transferred ROR2 remains active. Wnt/PCP signals via JNK to control epithelial cell polarity in *Drosophila* (23) and morphogenetic movements in vertebrates (24). Thus, we used a JNK signaling reporter system to quantify the Wnt/PCP response in receiving cells. To decipher the response of AGS cells to transferred ROR2 (rather than endogenous receptor), a ROR2 knockout AGS cell line was produced, which was subsequently transfected with the JNK-KTR-mCherry reporter plasmid, and a stable clone was selected. This cell line (AGS-B18) was used as the receiving cells, in which the reporter translocates from the nucleus to the cytoplasm upon activation of the JNK signaling pathway (Fig. 4*A*). The pCAF2 cell line was used as the producing cell in consequent experiments, as they have similar expression levels of ROR2 as the primary gCAM but survive longer and are genetically and phenotypically constant. pCAF2 cells were transfected with either a membrane marker (WT pCAF2), ROR2, or a dominant-negative ROR2 lacking the intracellular tyrosine kinase domain (ΔCD-ROR2). These were cocultured with AGS-B18 cells for 24 h and then imaged using confocal microscopy. Receiving cells for downstream analysis were determined as those in direct contact with transfected pCAF2 cytonemes (Fig. 4*B*). Cells which show no obvious cytoneme contact have been excluded from the analysis, as it was unclear whether they have been in contact with the CAF-filopodial network at an earlier time point. Quantification of the cytoplasmic/nuclear ratio of reporter protein indicated that the JNK signaling pathway is activated in receiving AGS cells upon coculture with both WT pCAF2 and pCAF2 overexpressing ROR2 (Fig. 4 *B* and *C*). However, JNK signaling is significantly decreased in receiving AGS cells that are cocultured with pCAF2 cells expressing ΔCD-ROR2. To further examine the contribution of the transferred ROR2 to the induction of JNK signaling in the receiving cell, we measured the mean fluorescence of ROR2-BFP in the receiving cells. Receiving cells were included for analysis in this subset when the transferred ROR2 was quantifiable and the JNK reporter activity was within a linear range. Simple linear regression was used to test whether the amount of ROR2 transferred significantly predicted the ratio of JNK localization (*SI Appendix*, Fig. S4*B*). The overall regression was statistically significant [R^2^ = 0.314, F (df regression, df residual) = 9.618, *P* = 0.0054], suggesting a direct correlation between transferred ROR2 and JNK signaling activation.

To further probe the paracrine signaling activity of transferred ROR2, we used AGS cells as the producing and receiving cell line because AGS display a very low expression of both ROR2 and WNT5A compared to pCAF2 cells (Fig. 1). Coculture of the AGS-B18 cells with wild-type AGS cells or WNT5A transfected AGS cells does not lead to a significant increase of JNK signaling in the receiving cells (Fig. 4*D*), supporting the importance of ROR2 as a key receptor for WNT5A. Next, we transfected the producing AGS cells with ROR2 or ROR2/WNT5A, leading to a significant increase in paracrine JNK activation, suggesting the requirement for ROR2 in paracrine Wnt/PCP activation. Furthermore, the amount of transferred ROR2-BFP was quantified in a subset of AGS-B18 cells cocultured with AGS transfected with ROR2 BFP, and we found a direct and significant correlation between transferred ROR2-BFP and JNK reporter activation in the receiving cells (*SI Appendix*, Fig. S4*C*, R^2^ = 0.1816, *P* = 0.0044). Importantly, paracrine JNK activation was ablated when the producing AGS cells were transfected with the dominant-negative dCD ROR2 (Fig. 4*D*). Finally, we transfected WNT5A and dCD-ROR2 in the producing cells leading to a similar inhibition of JNK signaling in the receiving AGS-B18. We conclude that active ROR2 receptors are transported—most likely on cytonemes—to the receiving cells to induce paracrine Wnt/PCP signaling. We performed further experiments to specifically investigate the role of cytonemes in this transfer and to determine the contribution of secreted complexes, for example, bound to exovesicles. Coculture of Wnt5A/ROR2-expressing AGS cells with AGS JNK reporter cells shows a significant increase of JNK signaling activation compared to the WT AGS/AGS reporter cells (Fig. 4*E*). Next, we cultured AGS-B18 reporter cells in media harvested from AGS cells transfected with ROR2 and WNT5A. We observed no significant activation of JNK signaling. Finally, we asked how signaling is altered when the producing cells cannot form cytonemes. To block cytoneme formation, we used the mutated form of IRSp53^4K^. We found that AGS cells transfected with this dominant negative form of IRSp53 and ROR2/WNT5A had significantly shorter cumulative filopodia length than AGS cells transfected with ROR2 and WNT5A alone (*SI Appendix*, Fig. S4*A*). Then, we cocultured the ROR2/WNT5A/dnIRSp53 transfected producing cells with the AGS-B18 reporter cell line. We observed no significant activation of JNK signaling in the receiving cell (Fig. 4*E*). These results indicate that cytoneme-mediated transport of ROR2/WNT5A complexes is the primary route of intercellular transfer, which cannot be compensated by secreted, EV-based mechanism.

### CAF-Produced ROR2 Induces Polarization of the Actin Cytoskeleton in Recipient AGS Cells.

**Fig. 5.**
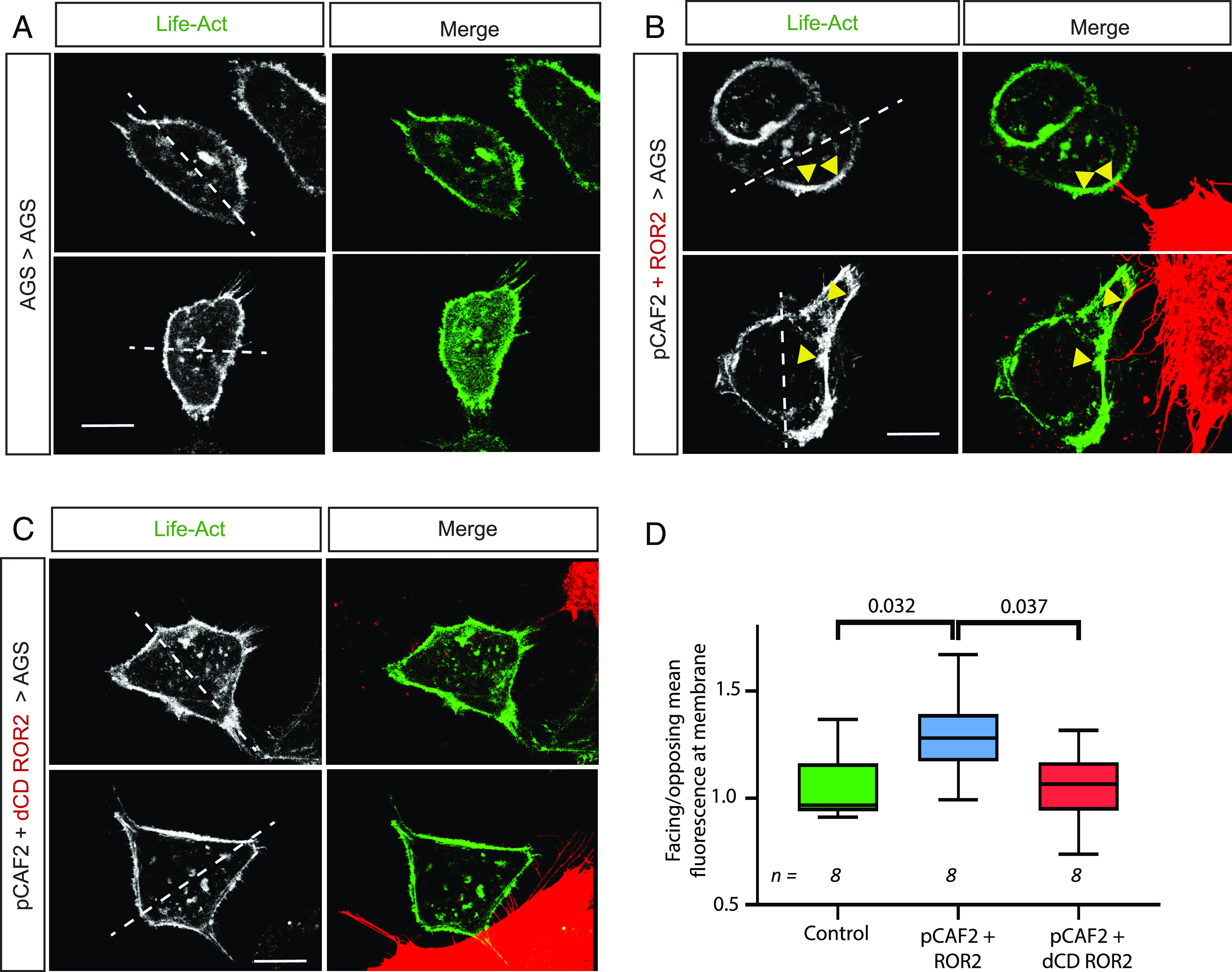
ROR2 induces actin polarization in receiving AGS cells. AGS cells were transfected with LifeAct GFP and cultured either (*A*) on their own (Control) or with pCAF2 cells overexpressing (*B*) ROR2 mCherry or (*C*) dCD ROR2 mCherry. Cells were imaged using confocal microscopy. The left-hand panel shows the 488-nm channel only (LifeAct-GFP), and the right-hand panel shows the merge of the green channel with the red channel to show the location of the producing cell and contact points. White dashed lines indicate a bisecting line through the center of the nucleus used to define the facing and opposing sides of the receiving cell. Yellow arrows indicate actin accumulation at contact points with ROR2-producing pCAF2 cells. The scale bar represents 10 μm. Two representative images from each condition are shown. (*D*) Quantification of facing/opposing mean fluorescent-labeled actin in receiving AGS cells in contact with cells as indicated on the *X* axis. n = number of receiving cells quantified. Significance was calculated using an unpaired *t* test between each two conditions.

Activation of JNK signaling via the Wnt/PCP pathway is associated with cell polarity and migration (26). To determine whether transferred ROR2 can affect polarity, we analyzed the actin distribution at the cell cortex of the receivers. Therefore, AGS cells were transfected with LifeAct-GFP and cocultured with pCAF2 cells expressing either ROR2 or ΔCD-ROR2. Individual AGS cells contacted by a pCAF2 cell were analyzed by a live-imaging time-lapse approach (Fig. 5 *A*–*C*). The ratio of fluorescent cortical actin on the facing side of the cell over the opposing side of the contact site was measured. AGS control cells show a homogeneous distribution of cortical actin (Fig. 5 *A* and *D*). However, we found a significant polarization of actin of AGS cells to the side in contact with pCAF2 cells expressing ROR2 (Fig. 5 *B* and *D*). We could not detect this phenotype in cells cocultured with pCAF2 cells expressing the dominant-negative ΔCD-ROR2 (Fig. 5 *C* and *D*).

Due to the complex nature of the pCAF2 filopodial network and the high mobility of the AGS cells, it was challenging to measure cell polarity in a simple coculture system. Therefore, we developed a bespoke PDMS microchamber to explore the effect of CAF-derived ROR2 on AGS polarization and migration. AGS and pCAF2 cells were cultured in chambers on either side of a series of diamond-shaped pillars, providing better separation and directionality to visualize the molecular interactions between the two populations of cells. Pillars with a dimension of 40 μm × 40 μm, and gaps of 4 μm retain the pCAF2 cells while allowing filopodial contacts to form and the AGS cells to migrate in two dimensions. AGS cells were transfected with LifeAct-GFP and cocultured in the PMDS microchambers with pCAF2 cells expressing ROR2 mCherry. In some cases, we observed a directional extension of protrusion toward the Ror2-expressing cells; however, due to insufficient data points, we report these results only as a proof-of-principle experiment, with the method shown and described (*SI Appendix*, Fig. S5).

### ROR2 Induces Directional Migration and Invasion in 3D Models.

To determine whether the ROR2-dependent induced polarization of individuals receiving AGS cells observed above resulted in the migration of whole populations of AGS cells, we utilized a standard 2D migration/wound healing assay. Surprisingly, we found that populations of AGS cells transiently expressing ROR2 and WNT5A had a larger remaining gap after 18 h of incubation than wild-type AGS cells (*SI Appendix*, Fig. S6 *A* and *B*). Upon close examination of time-lapse series of wild-type AGS cocultured with pCAF2 cells, we observed that AGS cells in close proximity to pCAF2 cells migrate within a narrow radius of the pCAF2 cell rather than migrating away and across the gap (*SI Appendix*, Fig. S6*C*). In contrast, AGS cells in the same field that did not start close to a pCAF2 cell migrated much further. This led us to hypothesize that the pCAF2 cells induce a directional, polarized migration in the AGS cells rather than a general increase in migratory capacity.

**Fig. 6.**
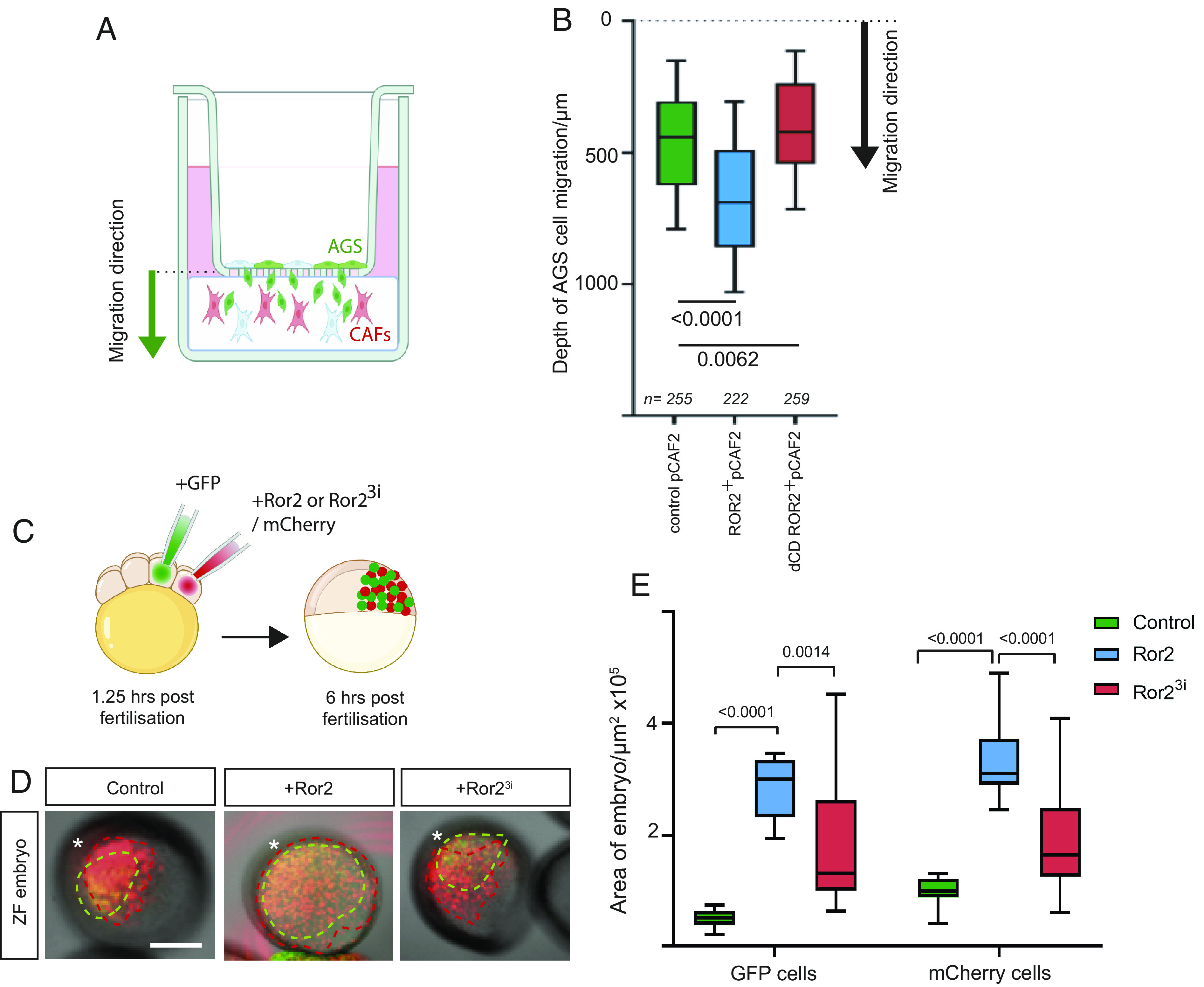
ROR2 induces polarized migration and invasion in 3D models. (*A*) Schematic demonstrating the 3D invasion assay. AGS cells transiently transfected with membrane GFP are cultured in a monolayer in transwells with an 8-μm pore size. pCAF2 cells transfected with either membrane mCherry, ROR2 mCherry, or dCD ROR2 mCherry are cultured in 3D using GrowDex at the bottom of the well, ensuring contact is made between the GrowDex and the transwell (*SI Appendix*, Fig. S6*D*). The black arrow indicates the migratory direction from the GFP-positive AGS cells into the hydrogel containing mCherry labeled CAFs. Following incubation for 72 h, the GrowDex is imaged using fluorescent light microscopy. Invading GFP-positive AGS cells are identified digitally by Imaris based on their size (20 μm in x, y, and z) to exclude cell debris and converted to dots (*SI Appendix*, Fig. S6*E*). The distance of each AGS cell away from the transwell is then calculated. (*B*) Depth into the hydrogel that GFP+ive AGS cells invade when cultivated with pCAF2 cells transfected with either membrane mCherry (pCAF2 control), ROR2 mCherry or dCD ROR mCherry as indicated on the *X* axis. include technical and biological repeats. The dashed line at the top of the graph represents the location of the transwell. Boxes represent 90% quartile, the centerline indicates the mean, and the whiskers indicate the range. n = number of AGS cells measured. Results from four experiments representing biological and technical repeats are shown. Significance was calculated using an unpaired *t* test. (*C*) Embryos were injected with membrane mCherry mRNA only (control) or plus Ror2 mRNA (Ror2) or Ror2^3i^ mRNA (Ror2^3i^) into one cell out of eight blastomeres, and the adjacent cell was injected with Gap43-GFP mRNA to generate two independent clones in the same embryo. (*D*) The live embryos were mounted and imaged at 6 h postfertilization, and the area of each embryo containing red and green-labeled cells was measured for each condition as a representation of how far cells from each clone (*) had migrated. Scale bar represents 200 µm. (*E*) Area of embryos containing GFP- or mCherry-positive cells indicated on the *X* axis for the different conditions (green bars: control—injected with membrane mCherry alone, blue bars: injected with Ror2 mRNA and membrane mCherry, red bars: injected with Ror2^3i^ and membrane mCherry). Boxes represent 95% quartile, the centerline indicates the mean, and whiskers indicate the range. Significance was calculated using ordinary one-way ANOVA with Tukey’s multiple comparison test.

To test this, we established an advanced 3D invasion assay (Fig. 6*A*). In this assay, pCAF2 transfected with either ROR2 or ΔCD-ROR2 were cultured in GrowDex hydrogel in 96-well plates, and AGS cells transfected with membrane GFP were cultured in a monolayer in a transwell plate placed on top of, and in contact with, the hydrogel. This hydrogel was selected for use as it is consistent between batches and, unlike Matrigel, does not contain cancerous microenvironment factors. The GrowDex was seeded with a high density of pCAF2 cells, which secrete ECM components to model the tumor microenvironment in a controlled manner. We confirmed that our AGS and pCAF2 cells grew normally in this 3D culture system and formed filopodial contacts (Fig. 1*B*). We next determined that the AGS cells would migrate through the transwells and into the pCAF2 containing hydrogel (*SI Appendix*, Fig. S6*D*). Following incubation for 72 h, the wells were imaged using fluorescent light microscopy. Images were analyzed and quantified using Imaris cell imaging software. AGS cells were identified in the hydrogel as GFP-positive cells with a diameter in x, y, and z of more than 20 μm. Visual inspection of overlaid green and bright-field channels confirmed that this detection method corresponded to cells rather than debris or background. GFP-positive AGS cells were converted to dots, as shown in *SI Appendix*, Fig. S6*E*, and the depth that individual AGS cells migrated into the hydrogels was measured using Imaris. AGS cells migrated significantly less distance into the hydrogel containing pCAF2 cells expressing ΔCD-ROR2 than either wild-type pCAF2 cells or pCAF2 cells overexpressing ROR2 (Fig. 6*B*). This adds further evidence to support our hypothesis that the pCAF2 cells provide a ROR2-dependent signal to the AGS cells to invade and migrate in a directional fashion. It is important to mention that also the usage of 3D models can resemble some cellular behavior; the mechanism might differ within a complex tumor environment.

Next, we performed an in vivo migration experiment in zebrafish to further support our idea that ROR2-expressing cells increase the directional migration of the neighboring cells. Therefore, we generated two independent clones in the same embryo. Wild-type zebrafish embryos were injected with membrane *mCherry* mRNA ± *ror2* mRNA into one cell out of eight blastomeres, and the adjacent cell was injected with *Gap43-GFP* mRNA. (Fig. 6*C*). The live embryos were mounted and imaged at 6 h postfertilization (hpf) (Fig. 6*D*). The area of the embryo containing red and green cells was measured. mCherry-expressing cells migrated over a greater area of the embryo when coexpressing Ror2, suggesting that autocrine PCP signaling drives this migratory process (Fig. 6*E*). This effect was ablated when the mCherry cells coexpressed dominant negative Ror2^3i^. Interestingly, the GFP-expressing cells also migrated over a greater area of the embryo when mCherry plus Ror2 was expressed in the adjacent cell population compared to embryos containing clones of mCherry alone cells or mCherry plus Ror2^3i^ (Fig. 6*D*). This result indicates that the GFP-expressing cells are induced to migrate further, presumably via paracrine transfer of ROR2 from adjacent cells.

Collectively, these results indicate that the transfer of the ROR2 receptor from CAFs to gastric cancer cells increases the capacity of the receiving tumor cell to respond to Wnt/PCP signaling—even in the absence of endogenous ROR2 receptors. Furthermore, transferred and active ROR2 receptors affect polarity and directional migration in the cancer cell and increase migration and invasion in 3D in vitro and in vivo models.

## Discussion

The aberrant expression of WNT5A in intestinal tumor microenvironments is well documented (27, 28). Still, the question of how cancer cells respond to PCP signaling is perplexing, given the downregulation of key receptors such as the concomitant coreceptor ROR2 in many gastric tumors. Here, we show that the WNT5A coreceptor ROR2 can be transferred from CAFs to gastric cancer cells via signaling filopodia, thereby inducing JNK signaling, actin polarization, and directional migration in the receiving cell. This unexpected intercellular signaling mechanism of receptor transfer provides insights and a possible explanation of how gastric cancer cells respond to high WNT5A in their environment.

Frequent upregulation of *WNT5A* mRNA has been previously described in primary gastric cancer (10), and WNT5A has been shown to be the only noncanonical Wnt ligand that is consistently up-regulated in gCAM (11). We confirmed the overexpression of WNT5A in gCAM. In addition, we report that WNT5A is equally highly expressed in pancreatic CAFs. In parallel to WNT5A, we observe an upregulation of ROR2 in gCAM/pCAF. In support of this finding, bone marrow–derived mesenchymal stem cells were also shown to have significantly higher ROR2 mRNA than the gastric cancer cell line MKN45 (29). Moreover, high ROR2 expression was identified in the stroma of 43% of pancreatic ductal adenocarcinoma (PDAC) tissues. However, this was not significantly different from matched tissues or benign pancreatic lesions, and the stromal cell type was not characterized (30). In addition, high stromal ROR2 expression was significantly associated with lymph node invasion and tumor stage in this study.

The key finding presented here is that a membrane-spanning protein, the receptor ROR2, can be transported from CAFs to signal-receiving gastric cancers via filopodia to allow the gastric cancer cell to respond to ligands such as WNT5A, a paradigm shift in our understanding of how cells can respond to paracrine Wnt signaling. We have previously shown that Wnt ligands can be transported intercellularly over long distances via specialized actin-rich signaling filopodia, also known as cytonemes (19, 20, 31). More recently, we have shown that Wnt3 is transported intercellularly via cytonemes and facilitates tumor progression in gastric cancer (18). Therefore, cytoneme-mediated intercellular transport of Wnt components provides an attractive hypothesis to describe how long-range signaling may be achieved in the tumor microenvironment. In addition to the Wnt ligands, we observe fluorescently labeled, overexpressed ROR2 in receiving AGS cells over considerable time periods and in most directly contacted cells. Despite being a striking observation of a cytoneme-mediated Wnt receptor transfer to a signal-receiving cell, there is some precedent for intercellular receptor trafficking. Evidence suggests that exovesicles can play a role in receptor transfer, including the transport of Fzd10 mRNA in gastric and colorectal cancer (32), and intercellular transfer of the oncogenic receptor EGFRvIII by microvesicles derived from glioma cells has been reported (33). Although we do not rule out the possibility of exosome-mediated intercellular transfer of ROR2 in the findings presented here, we observe that paracrine JNK signaling correlates with the relative amount of producing cell–derived ROR2 in the receiving AGS cell. The receiving cells were included in the analysis only if cytoneme-based contact could be identified, which provides evidence for a more direct role in the cell-to-cell transfer of activated ROR2. Moreover, reporter cells cultured in conditioned media from AGS cells transfected with ROR2 and WNT5A showed a significant reduction in JNK activation in the receiving population compared to a coculture system with the same producing and receiving cells. Similarly, AGS reporter cells cocultured with producing cells expressing a dominant negative form of IRSp53 to reduce the filopodial length in addition to ROR2 and WNT5A also showed a significant reduction in JNK activation in the receiving cells. Taken together, these results indicate that cytoneme-mediated transport of receptor–ligand complexes is the primary mechanism of transfer in this system as opposed to secreted factors.

In addition, there is evidence that other receptors can be transported along cytonemes. Thickveins (the receptor for Decapentaplegic, DPP) and EGFR has been observed to be moving in puncta along cytonemes in *Drosophila* (34, 35). Moreover, confocal fluorescent microscopy of GFP-tagged FZD7 shows retrograde transport along cytonemes during chicken embryonic somite development (36). We have previously shown that ROR2 also colocalizes with other ligands and components of the Wnt signaling pathway on cytonemes (18, 19, 31), including the canonical ligand Wnt8a. This raises the intriguing question of how the trafficking of different proteins to the cytoneme tip for transport is regulated and what molecular mechanisms determine which filopodia become Wnt-carrying cytonemes.

Overexpressed ROR2 was transferred via cytonemes in association with producing cell membranes, and high-resolution imaging leads us to suggest that the tip of the cytoneme buds off following contact with a receiving cell and is endocytosed. In support of this hypothesis, we found producing cell membrane colocalizing with ROR2 in the receiving cell and demonstrated the involvement of Dyn2 in the endocytosis process. We also report the presence of both VAMP3 and VAMP8 at cytoneme tips. A role for SNARE proteins in cytoneme-mediated transport of signaling components is not unprecedented, as a mechanism mediated by SNARE proteins is required for Patched (Ptc) transport by cytonemes in the *Drosophila* wing disc (22). However, here, colocalization of SNARE proteins with Wnt ligands has been identified, and this raises interesting questions regarding the similarities between cytoneme-based transport of different proteins. Moreover, it may provide avenues of investigation as to how target cells are identified by cytonemes and how the contact site is initiated and maintained.

In addition to the overexpression experiments, we show that the transfer of ROR2 complexes also happens at the endogenous level between primary gCAM and AGS cells and that transferred ROR2 is endocytosed in receiving cells. Most importantly, by using a ROR2 antibody conjugated to a pH-dependent dye, we are able to determine the orientation of the receptor on the cytoneme and in the endosome, such that the CRD domain is exposed to the extracellular side and, subsequently, facing into the lumen of the vesicle (Fig. 3). Consequently, the kinase domain is orientated toward the cytoplasm of the cytoneme and the cytoplasm of the receiving cell. This orientation allows continuous signaling into the producing cell and, after handover, signaling into the receiving cell. Indeed, our studies reveal that pCAF2-derived, paracrine transport of ROR2 can induce JNK signaling in AGS-receiving cells at a level that directly correlates with the amount of functional ROR2 transported.

Previous studies have shown a role for MSC-derived ROR2 and WNT5A in autocrine induction of the C-X-C Motif Chemokine Ligand CXCL16, which in turn promotes the proliferation of MKN45 gastric cancer cells via CXCR6 (29). In addition, the knockdown of CXCL16 in MSC suppressed the migration of MKN45 cells (37). However, bone marrow-derived MSC cells are a heterogenous pool of progenitor cells, and here, we report specifically on the function of ROR2 in CAFs. Moreover, the knockdown of ROR2 in MSC had a greater inhibitory effect on MKN45 migration than the knockdown of CXCL16, suggesting a role for ROR2 supplementary to its role in CXCL16 upregulation. Furthermore, when AGS cells transfected with ROR2 and WNT5A are used as the signal-producing cells in our study, we report a similar level of paracrine JNK activation in the receiving AGS cells as when pCAF2 cells are the signal-producing cells. In both, this experiment and in our parallel zebrafish model, it is unlikely that the same panel of chemokines and other soluble signals would be present. There are no obvious orthologues of CXCL16 or CXCR6 in zebrafish (38), although CXCL chemokines have been shown to have a role in migration at later stages of zebrafish embryonic development. In addition, blocking WNT5A-mediated ROR2 internalization suppressed gastric cancer tumor cell invasion and metastasis in WNT5A-high but not WNT5A-low cells (39), suggesting a direct role for ROR2 in activating the Wnt/PCP pathway rather than a chemokine signaling pathway. It has also been shown that filopodia formation mediated by ROR2 is required for WNT5A-induced cell migration in human melanoma cell lines (40) further supporting our observation of a role for direct transport of ROR2 in inducing migration in AGS cells.

In support of our model, we observed a similar phenomenon occurring in zebrafish development and cytonemes or intercellular bridges could facilitate the intercellular transport in the embryo (20, 41); however, this needs to be investigated in the future. In a parallel study by our group, quantitative fluorescent imaging via fluorescent correlation spectroscopy (FCS) and FLIM-FRET was developed to provide evidence that zebrafish Ror2 and the relevant ligand Wnt5b are significantly correlated in the producing cell, on cytoneme tips and also in the receiving cell (42). Taken together, we suggest a conserved and essential role for this signaling mechanism in both vertebrate embryogenesis and disease.

Our data show that ROR2 is an important receptor for WNT5A. Using various in vitro and in vivo assays, we demonstrate a promoting role of transferred ROR2 in establishing cell polarity and enhancing the invasion of gastric cancer cells. In support of our data, enhanced levels of WNT5A facilitate cell migration and invasion of gastric cancer cells (43). Similarly, WNT5A regulates cellular migration and invasion in various types of colon cancers with a dependency on ROR2 function (44). Here, WNT5A promotes the formation of adhesion sites leading to directional migration, and this activity is reduced in Wnt5a knockdown colon cancer cells leading to reduced directional migration. WNT5A can also induce polarization of tumor-associated macrophages (TAMs) and ultimately promoting tumor growth and metastasis of colorectal cancers (45). However, it is unclear whether TAMs produce sufficient levels of ROR2 to respond to WNT5A or whether they rely on CAF-transferred ROR2 similar to tumor cells.

In conclusion, we show that the Wnt/PCP coreceptor ROR2 can be directly transported from CAFs to AGS cells, thereby inducing JNK signaling, actin polarization, and directional migration in the receiving cell in a ROR2 dependent manner. These observations were confirmed in an in vivo zebrafish model of cell migration reported in this paper and further supported by a parallel developmental zebrafish study that validates this Wnt/PCP signaling mechanism. However, further experiments are required to support this mechanistic finding in a complex human tumor environment. Taken together, these results increase our understanding of how gastric cancer cells can respond to their microenvironment and the molecular mechanisms that promote migration and metastasis.

## Materials and Methods

### Cell Culture.

The human gastric adenocarcinoma cell line AGS was purchased from ECACC and maintained in Roswell Park Memorial Institute (RPMI) 1640 Medium supplemented with 10% fetal bovine serum (FBS) (Fisher). Fibroblast cell lines were generously gifted by Melissa Fishel of IU Simon Cancer Research Center and isolated as described in ref. 46. Fibroblast nomenclature was reduced for purposes of simplicity with “pCAF2” referring to UH1303-02 cells. pCAF2 cells were maintained in Dulbecco’s modified eagle medium (DMEM) supplemented with 10% fetal bovine serum. Human primary gastric myofibroblasts were generously gifted by Andrea Varro and are as described in ref. 47. Gastric myofibroblasts were cultured in DMEM with L-glutamine containing 10% fetal bovine serum, 1% modified Eagle medium nonessential amino acid solution, 1% penicillin/streptomycin, and 2% antibiotic–antimycotic. The medium was replaced routinely every 48 to 60 h, and cells were passaged at confluence up to 10 times. 3D cell culture was performed using GrowDex® hydrogel (UPM). The stable ROR2 knockout JNK reporter AGS cell line was generated by transfection first with the validated ROR2 CRISPR plasmids sc-401324 (Santa Cruz Biotechnology). ROR2 knockout clones were confirmed by sequencing, and the B18 clone was transfected with the JNK KTR-mCherry plasmid (25, 48), followed by selection with blasticidin. A stable clone was selected, which expressed the JNK reporter at a level suitable for detection by confocal microscopy. All cells were maintained at 37 °C with 5% CO_2_.

### Plasmids and Transfection.

All plasmids used are listed in *SI Appendix*. Transfections were performed using a 4D-Nucleofector Unit (Lonza), with the P2 Primary Cell Kit for pCAF2 cells and the SF Cell Line kit for AGS cells. AGS cells were additionally transfected with Fugene HD Transfection Reagent (Promega) if high transfection efficiency was not critical.

### qPCR.

RNA for qPCR was collected from cell pellets using the QIAGEN RNeasy kit according to the manufacturer’s instructions. qRT-PCR was then performed using the SensiFAST™ SYBR® Lo-ROX One-Step Kit with half volumes according to the manufacturer’s protocol and run using Applied Biosystems QuantStudio6 Flex. Primer sequences are listed in *SI Appendix*.

### Immunofluorescence.

Cells were plated onto glass coverslips and following 24-h incubation were washed in 1×PBS and fixed using modified MEM-Fix (4% formaldehyde, 0.25 to 0.5% glutaraldehyde, 0.1 M Sorenson’s phosphate buffer, pH7.4) (21, 49) for 7 min at 4 °C. Cells were then incubated in permeabilization solution (0.1% TritonX-100, 5% serum, 0.3 M glycine in 1×PBS) for 1 h at RT. Primary antibodies (mouse anti-Ror2: Santa Cruz H-1, rabbit anti-Wnt5a/b: ProteinTech 55184-1-AP) were diluted in incubation buffer (0.1% Tween20, 5% serum in 1×PBS) and coverslips incubated in 50 µl spots on parafilm overnight at 4 °C. Coverslips were then washed with 1×PBS 3× for 5 min before incubation in 50-µL spots of secondary antibodies (goat anti-mouse AF647: Abcam ab150115, goat anti-rabbit AF405: abcam 175652) diluted in incubation buffer for 1 h at RT. Coverslips were then washed 3× for 5 min with 1×PBS before mounting onto glass slides using ProLong Diamond anti-fade mountant (Invitrogen) and left to dry for 24 h before imaging. Confocal microscopy for immunofluorescent antibody imaging was performed on a Leica TCS SP8 laser-scanning microscope using the 63× water or oil objectives.

### Antibody Internalization Assay.

Anti-Ror2 antibody (R&D Systems, AF2064) or Normal Goat IgG control sera (R&D systems) were labeled with pH-sensitive pHrodo™ Green STP ester dye (Invitrogen) as per the manufacturer’s instructions. Labeled antibodies were purified using Micro Bio-Spin™ P-30 Gel Columns (Biorad). Populations of cells as required were cocultured for 24 h to allow adherence, and then 3 nM labeled antibody or control sera in Phenol-red free media were added for 20 h before cells were imaged on a Leica TCS SP8 confocal microscope using a 63× oil objective.

### JNK Reporter Assay.

AGS *ROR2* KO cells stably expressing JNK-KTR-mCherry (AGS-B18) were cocultured with either pCAF2 cells or AGS cells transfected with indicated plasmids for 24 h. For conditioned media experiments, AGS cells were transfected with ROR2 and WNT5a and incubated for 24 h. Media were collected, centrifuged for 10 min at 5,000 rpm to remove cells, and added to reporter AGS B18 cells. After 12 h, a further batch of conditioned media was collected from the producing cells and used to replace the media on the AGS B18 reporter cells. Cells were imaged after a total of 24 h in conditioned media, consistent with the time for the coculture experiments. Cells were imaged on a Leica TCS SP8 confocal microscope using a 40× oil objective. Fluorescence intensity of each receiving cell’s cytoplasm and nucleus was measured using Fiji software, and the cytoplasmic:nuclear ratio (C:N) was calculated.

### Actin Polarization.

AGS cells were transfected with LifeAct-GFP and incubated for 24 h for recovery. pCAF2 cells were transfected as required and also incubated for 24 h recovery. Cells were trypsinized, counted, and the two populations cocultured for 24 h. Cells were imaged using a Leica TCS SP8 laser-scanning microscope using the 63× oil objective. The fluorescence intensity was analyzed in AGS cells that were in contact with a pCAF2 cell using FIJI. A line was drawn through the middle of the nucleus of the receiving AGS cell to define facing and opposing sides, and the mean fluorescence at the membrane of each side was measured.

### 3D Invasion Assay.

pCAF2 cells were transfected as required and cultured for 24 h for recovery. AGS cells were transfected with pCS2+ Gap43-GFP (membrane-GFP) and also incubated for 24 h. The 3D invasion assays were performed using GrowDex® hydrogel (UPM) as the matrix supporting 3D cell growth. For the experiment, 1 × 10^5^ pCAF2 cells were resuspended in 40 µL complete cell culture medium (DMEM + 5% FBS, minus phenol red), and then mixed with 90 µL of the 1.0% hydrogel stock to achieve a 0.75% w/v hydrogel solution. Then, 80 µL of this suspension was dispensed to a well of a 96-well transwell plate (Corning) using a wide-bore pipette. The insert was placed on top, and 5 × 10^3^ transfected AGS cells aliquoted in 100 µL of DMEM + 5% FBS minus phenol red on top of the 8-µm pore-size membrane. Media were exchanged once per day for 72 h, and on the third day, cells were imaged using a Leica DMI6000 light microscope with a 20× dry objective. One-millimeter stacks were obtained, with a frame at every 5 µm. Images were analyzed using Imaris, with green AGS cells converted to spots and the depth of their position in the hydrogel measured. Three stacks were obtained in each well, and the experiments were performed in triplicate.

### In Vivo Zebrafish Migration Assay.

WIK wild-type zebrafish (*Danio rerio*) were maintained at 28 °C and on a 14-h light/10 h dark cycle (50). Zebrafish care and all experimental procedures were carried out in accordance with the European Communities Council Directive (2010/63/EU), Animals Scientific Procedures Act 1986 and under personal and project licenses granted by the UK Home Office, and ethically approved by the Animal Welfare and Ethical Review Body at the University of Exeter. Embryos were injected with membrane mCherry mRNA ± Ror2 mRNA into one cell out of eight blastomeres, and the adjacent cell was injected with Gap43-GFP mRNA to generate two independent clones in the same embryo. The live embryos were mounted and imaged at 6 h postfertilization using a Leica TCS SP8 confocal microscope, using 10× dry objective. The area of the embryo containing red and green cells was determined using FIJI. The ordinary one-way ANOVA together with Tukey’s multiple comparisons test was performed using GraphPad Prism 9.0.

## Supplementary Material

Appendix 01 (PDF)

Movie S1. The movie shows a rotation of a gCAM cell transfected with membrane GFP extending multiple filopodia in a 3D culture that contact AGS cells transfected with membrane mCherry, and transport multiple membrane-bound vesicles from producing to receiving cell (see also Fig. 1b and Fig. S1a).

## Data Availability

All study data are included in the article and/or supporting information.

## References

[1] R. Nusse, H. Clevers, Wnt/beta-catenin signaling, disease, and emerging therapeutic modalities. Cell **169**, 985–999 (2017). 28575679 10.1016/j.cell.2017.05.016

[2] K. VanderVorst, J. Hatakeyama, A. Berg, H. Lee, K. L. Carraway IIIrd., Cellular and molecular mechanisms underlying planar cell polarity pathway contributions to cancer malignancy. Semin Cell Dev. Biol. **81**, 78–87 (2018). 29107170 10.1016/j.semcdb.2017.09.026 PMC5934344

[3] S. Stricker, V. Rauschenberger, A. Schambony, ROR-family receptor tyrosine kinases. Curr. Top Dev. Biol. **123**, 105–142 (2017). 28236965 10.1016/bs.ctdb.2016.09.003

[4] S. Rogers, S. Scholpp, Vertebrate Wnt5a - At the crossroads of cellular signalling. Semin. Cell Dev. Biol. **125**, 3–10 (2022). 34686423 10.1016/j.semcdb.2021.10.002

[5] I. Oishi , The receptor tyrosine kinase Ror2 is involved in non-canonical Wnt5a/JNK signalling pathway. Genes Cells **8**, 645–654 (2003). 12839624 10.1046/j.1365-2443.2003.00662.x

[6] Y. Minami, I. Oishi, M. Endo, M. Nishita, Ror-family receptor tyrosine kinases in noncanonical Wnt signaling: Their implications in developmental morphogenesis and human diseases. Dev. Dyn **239**, 1–15 (2010). 19530173 10.1002/dvdy.21991

[7] M. Enomoto , Autonomous regulation of osteosarcoma cell invasiveness by Wnt5a/Ror2 signaling. Oncogene **28**, 3197–3208 (2009). 19561643 10.1038/onc.2009.175

[8] M. P. O’Connell , The orphan tyrosine kinase receptor, ROR2, mediates Wnt5A signaling in metastatic melanoma. Oncogene **29**, 34–44 (2010). 19802008 10.1038/onc.2009.305 PMC2803338

[9] D. Ren, Y. Minami, M. Nishita, Critical role of Wnt5a-Ror2 signaling in motility and invasiveness of carcinoma cells following Snail-mediated epithelial-mesenchymal transition. Genes Cells **16**, 304–315 (2011). 21342370 10.1111/j.1365-2443.2011.01487.x

[10] T. Saitoh, T. Mine, M. Katoh, Frequent up-regulation of WNT5A mRNA in primary gastric cancer. Int. J. Mol. Med. **9**, 515–519 (2002). 11956659

[11] L. Wang , Distinct miRNA profiles in normal and gastric cancer myofibroblasts and significance in Wnt signaling. Am. J. Physiol. Gastrointest Liver Physiol. **310**, G696–704 (2016). 26939869 10.1152/ajpgi.00443.2015 PMC4867324

[12] P. Astudillo, A non-canonical Wnt signature correlates with lower survival in gastric cancer. Front Cell Dev. Biol. **9**, 633675 (2021). 33869179 10.3389/fcell.2021.633675 PMC8047116

[13] L. Yan, Q. Du, J. Yao, R. Liu, ROR2 inhibits the proliferation of gastric carcinoma cells via activation of non-canonical Wnt signaling. Exp. Ther. Med. **12**, 4128–4134 (2016). 28101190 10.3892/etm.2016.3883 PMC5228286

[14] L. M. Machesky, A. Li, Fascin: Invasive filopodia promoting metastasis. Commun. Integr. Biol. **3**, 263–270 (2010). 20714410 10.4161/cib.3.3.11556 PMC2918773

[15] T. Iguchi , Fascin expression in progression and prognosis of hepatocellular carcinoma. J. Surg. Oncol. **100**, 575–579 (2009). 19697358 10.1002/jso.21377

[16] A. Daponte , Prognostic significance of fascin expression in advanced poorly differentiated serous ovarian cancer. Anticancer Res. **28**, 1905–1910 (2008). 18630479

[17] C. Zhang, S. Scholpp, Cytonemes in development. Curr. Opin. Genet Dev. **57**, 25–30 (2019). 31404787 10.1016/j.gde.2019.06.005 PMC6838781

[18] D. Routledge, S. Rogers, H. Ashktorab, T. J. Phesse, S. Scholpp, The scaffolding protein Flot2 regulates cytoneme-based transport of Wnt3 in gastric cancer. bioRxiv [Preprint] (2022). 10.1101/2022.01.07.475396 (Accessed 8 January 2022). PMC9457691 36040316

[19] L. Brunt , Vangl2 promotes the formation of long cytonemes to enable distant Wnt/beta-catenin signaling. Nat. Commun. **12**, 2058 (2021). 33824332 10.1038/s41467-021-22393-9 PMC8024337

[20] E. Stanganello , Filopodia-based Wnt transport during vertebrate tissue patterning. Nat. Commun. **6**, 5846 (2015). 25556612 10.1038/ncomms6846

[21] S. Rogers, S. Scholpp, Preserving cytonemes for immunocytochemistry of cultured adherent cells. Methods Mol. Biol. **2346**, 183–190 (2021). 32803539 10.1007/7651_2020_305

[22] L. Gonzalez-Mendez , Polarized sorting of patched enables cytoneme-mediated Hedgehog reception in the Drosophila wing disc. EMBO J. **39**, e103629 (2020). 32311148 10.15252/embj.2019103629 PMC7265244

[23] M. Boutros, N. Paricio, D. I. Strutt, M. Mlodzik, Dishevelled activates JNK and discriminates between JNK pathways in planar polarity and wingless signaling. Cell **94**, 109–118 (1998). 9674432 10.1016/s0092-8674(00)81226-x

[24] H. Yamanaka , JNK functions in the non-canonical Wnt pathway to regulate convergent extension movements in vertebrates. EMBO Rep. **3**, 69–75 (2002). 11751577 10.1093/embo-reports/kvf008 PMC1083927

[25] S. Regot, J. J. Hughey, B. T. Bajar, S. Carrasco, M. W. Covert, High-sensitivity measurements of multiple kinase activities in live single cells. Cell **157**, 1724–1734 (2014). 24949979 10.1016/j.cell.2014.04.039 PMC4097317

[26] Y. Yang, M. Mlodzik, Wnt-Frizzled/planar cell polarity signaling: Cellular orientation by facing the wind (Wnt). Annu. Rev. Cell Dev. Biol. **31**, 623–646 (2015). 26566118 10.1146/annurev-cellbio-100814-125315 PMC4673888

[27] T. Hirashima , Wnt5a in cancer-associated fibroblasts promotes colorectal cancer progression. Biochem. Biophys. Res. Commun. **568**, 37–42 (2021). 34175688 10.1016/j.bbrc.2021.06.062

[28] M. Kanzawa, S. Semba, S. Hara, T. Itoh, H. Yokozaki, WNT5A is a key regulator of the epithelial-mesenchymal transition and cancer stem cell properties in human gastric carcinoma cells. Pathobiology **80**, 235–244 (2013). 23615002 10.1159/000346843

[29] G. Takiguchi, M. Nishita, K. Kurita, Y. Kakeji, Y. Minami, Wnt5a-Ror2 signaling in mesenchymal stem cells promotes proliferation of gastric cancer cells by activating CXCL16-CXCR6 axis. Cancer Sci. **107**, 290–297 (2016). 26708384 10.1111/cas.12871 PMC4814243

[30] J. Huang , High ROR2 expression in tumor cells and stroma is correlated with poor prognosis in pancreatic ductal adenocarcinoma. Sci. Rep. **5**, 12991 (2015). 26259918 10.1038/srep12991 PMC4531333

[31] B. Mattes , Wnt/PCP controls spreading of Wnt/beta-catenin signals by cytonemes in vertebrates. Elife **7**, e36953 (2018). 30060804 10.7554/eLife.36953 PMC6086664

[32] M. P. Scavo , FZD10 carried by exosomes sustains cancer cell proliferation. Cells **8**, 777 (2019). 31349740 10.3390/cells8080777 PMC6721576

[33] K. Al-Nedawi , Intercellular transfer of the oncogenic receptor EGFRvIII by microvesicles derived from tumour cells. Nat. Cell Biol. **10**, 619–624 (2008). 18425114 10.1038/ncb1725

[34] F. Hsiung, F. A. Ramirez-Weber, D. D. Iwaki, T. B. Kornberg, Dependence of Drosophila wing imaginal disc cytonemes on Decapentaplegic. Nature **437**, 560–563 (2005). 16177792 10.1038/nature03951

[35] S. Roy, H. Huang, S. Liu, T. B. Kornberg, Cytoneme-mediated contact-dependent transport of the Drosophila decapentaplegic signaling protein. Science **343**, 1244624 (2014). 24385607 10.1126/science.1244624 PMC4336149

[36] F. Sagar, C. Prols, M. Wiegreffe, Scaal, Communication between distant epithelial cells by filopodia-like protrusions during embryonic development. Development **142**, 665–671 (2015). 25617437 10.1242/dev.115964

[37] T. Ikeda , Mesenchymal stem cell-derived CXCL16 promotes progression of gastric cancer cells by STAT3-mediated expression of Ror1. Cancer Sci. **111**, 1254–1265 (2020). 32012403 10.1111/cas.14339 PMC7156785

[38] J. Bussmann, E. Raz, Chemokine-guided cell migration and motility in zebrafish development. EMBO J. **34**, 1309–1318 (2015). 25762592 10.15252/embj.201490105 PMC4491993

[39] H. Hanaki , An anti-Wnt5a antibody suppresses metastasis of gastric cancer cells in vivo by inhibiting receptor-mediated endocytosis. Mol. Cancer Ther. **11**, 298–307 (2012). 22101459 10.1158/1535-7163.MCT-11-0682

[40] M. Nishita , Filopodia formation mediated by receptor tyrosine kinase Ror2 is required for Wnt5a-induced cell migration. J. Cell Biol. **175**, 555–562 (2006). 17101698 10.1083/jcb.200607127 PMC2064592

[41] L. Caneparo, P. Pantazis, W. Dempsey, S. E. Fraser, Intercellular bridges in vertebrate gastrulation. PLoS One. **6**, e20230 (2011). 21647454 10.1371/journal.pone.0020230 PMC3102083

[42] C. Zhang, L. Brunt, S. Rogers, S. Scholpp, Cytoneme-mediated transport of active Wnt5b/Ror2 complexes is required for paracrine Wnt/PCP signaling in zebrafish gastrulation. bioRxiv [Preprint] (2022). 10.1101/2022.04.07.487468 (Accessed 8 April 2022).

[43] M. Kurayoshi , Expression of Wnt-5a is correlated with aggressiveness of gastric cancer by stimulating cell migration and invasion. Cancer Res. **66**, 10439–10448 (2006). 17079465 10.1158/0008-5472.CAN-06-2359

[44] E. R. Bakker , Wnt5a promotes human colon cancer cell migration and invasion but does not augment intestinal tumorigenesis in Apc1638N mice. Carcinogenesis **34**, 2629–2638 (2013). 23764752 10.1093/carcin/bgt215

[45] Q. Liu , Wnt5a-induced M2 polarization of tumor-associated macrophages via IL-10 promotes colorectal cancer progression. Cell Commun. Signal **18**, 51 (2020). 32228612 10.1186/s12964-020-00557-2 PMC7106599

[46] K. E. Richards , Cancer-associated fibroblast exosomes regulate survival and proliferation of pancreatic cancer cells. Oncogene **36**, 1770–1778 (2017). 27669441 10.1038/onc.2016.353 PMC5366272

[47] C. Holmberg , Release of TGFbetaig-h3 by gastric myofibroblasts slows tumor growth and is decreased with cancer progression. Carcinogenesis **33**, 1553–1562 (2012). 22610072 10.1093/carcin/bgs180 PMC3499060

[48] H. Miura, Y. Kondo, M. Matsuda, K. Aoki, Cell-to-cell heterogeneity in p38-mediated cross-inhibition of JNK causes stochastic cell death. Cell Rep. **24**, 2658–2668 (2018). 30184500 10.1016/j.celrep.2018.08.020

[49] W. J. Bodeen, S. Marada, A. Truong, S. K. Ogden, A fixation method to preserve cultured cell cytonemes facilitates mechanistic interrogation of morphogen transport. Development **144**, 3612–3624 (2017). 28827391 10.1242/dev.152736 PMC5665483

[50] M. Brand, M. Granato, C. Nüsslein-Volhard, Keeping and raising zebrafish. Zebrafish: A Practical Approach **1**, 7–37 (2002).

