# Supplementary material for: Cancer-associated fibroblasts influence Wnt/PCP signaling in gastric cancer cells by cytoneme-based dissemination of ROR2
Source: Proc Natl Acad Sci U S A. 2023 Sep 18;120(39):e2217612120. doi: 10.1073/pnas.2217612120 (PMC10523461; doi:10.1073/pnas.2217612120)
Supplement: Supplementary file 1 — Appendix 01 (PDF) [file pnas.2217612120.sapp.pdf]

PNAS

Supporting Information for

Cancer-associated fibroblasts influence Wnt/PCP signaling in gastric cancer cells by cytoneme-based dissemination of the receptor ROR2

Sally Rogers[1], Chengting Zhang[1], Vasilis Anagnostidis[1], Corin Liddle[2], Melissa Fishel[3], Fabrice Gielen[1], Steffen Scholpp[1]
[1] Living Systems Institute, University of Exeter, UK; [2] Bioimaging Centre, University of Exeter, UK[3] Indiana University, School of Medicine, Indianapolis, US

Corresponding author: Steffen Scholpp
Email: s.scholpp@exeter.ac.uk

This PDF file includes:

Supporting text, including Material and Methods
Figures S1 to S6
Legends for Movies S1

Supporting Information Text

Material and Methods.

Plasmids & Primers

The following plasmids were used in transfections: pCS2+ GPI-anchored mCherry (Membrane-mCh) (Scholpp et al., 2009), pCS2+ Gap43-GFP (membrane-GFP), pCS2+ Ror2^{3i} (Mattes et al., 2018), JNK KTR-mCherry (Regot et al., 2014), pCS2+ Ror2-mCherry (cloned in with ClaI and XbaI), pCS2+ Ror2-eBFP2 (cloned by inserting eBFP2 into pCS2+ Ror2 plasmid using XbaI and SnaBI), pCDNA Ror2-eBFP2 (Ror2 and eBFP2 amplified and cloned into pcDNA3.1 using the PmeI and NotI restriction sites), pcDNA dCDRor2-eBFP2 (Ror2 minus intracellular domain amplified and cloned into the pCDNA-eBFP2 backbone using NotI and XbaI restriction sites), pCS2+ Wnt5A-eGFP (Wnt5a cloned into a pCS2+ GFP backbone using ClaI and XbaI restriction sites), pCS2+ Wnt6a mCherry, IRSp534K-mCherry (Stanganello et al., 2015) VAMP3-GFP (Addgene 42310), VAMP8-GFP (Addgene 42311), RFP Dynamin2 K44A (Addgene 128153). Transfections were performed using a 4D-Nucleofector Unit (Lonza), with the P2 Primary Cell Kit for pCAF2 cells and the SF Cell Line Kit for AGS cells. AGS cells were additionally transfected with Fugene HD Transfection Reagent (Promega) if high transfection efficiency was not critical.

Primer sequences used were as follows: *ROR2* (Forward 5'- GTACGCATGGAACTGTGTGACG -3'); (Reverse 5'-AAAGGCAAGCGATGACCAGTGG -3'), *WNT5A* (Forward 5'-TACGAGAGTGCTCGCATCCTCA -3'); (Reverse 5'- TGTCTTCAGGCTACATGAGCCG-3'), *GAPDH* (Forward 5'-GTCTCCTCTGACTTCAACAGCG -3'); (Reverse 5'-ACCACCCTGTTGCTGTAGCCAA -3'). Relative mRNA expression was calculated using the $2^{-\Delta\Delta Ct}$ method.

Supplementary Figures

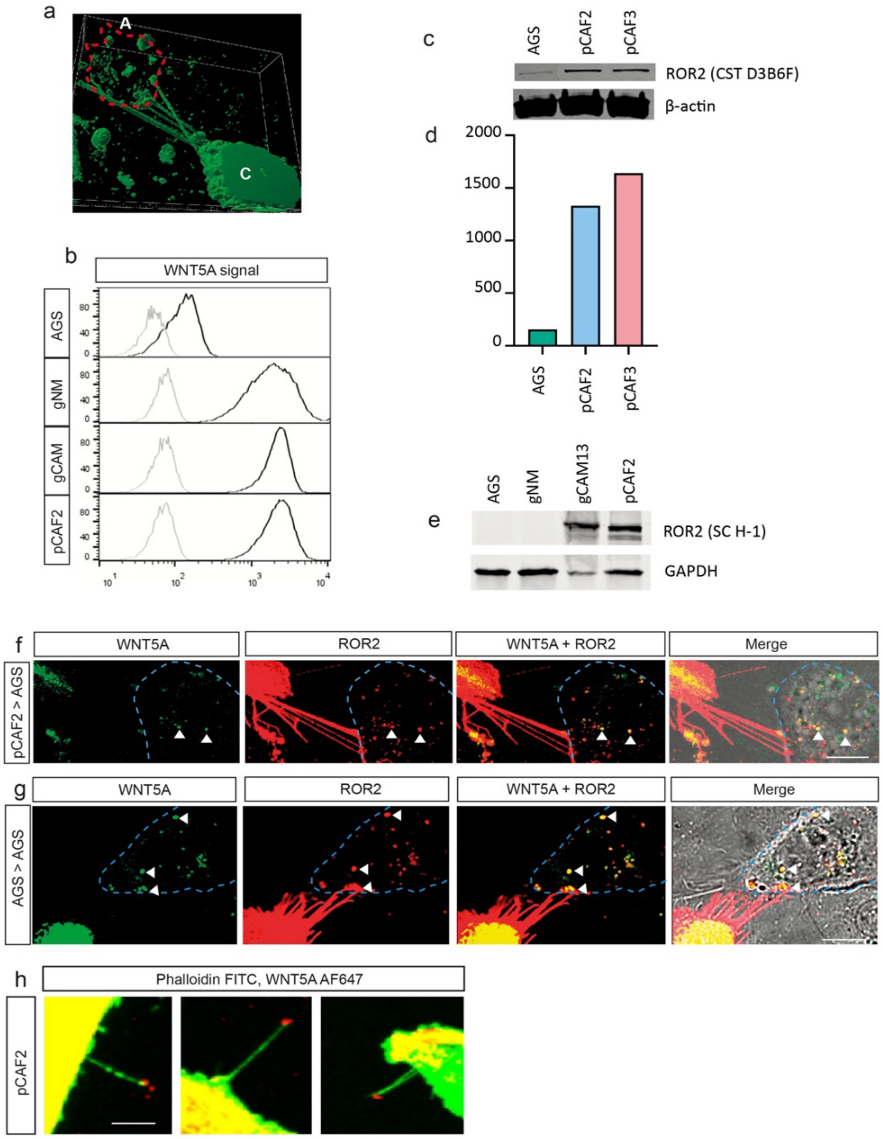

Figure S1. a. An alternative view from Fig. 1b showing the location of the membrane mCherry transfected AGS cell (A), and the membrane GFP transfected gCAM cell, indicated by C. **b.** Cell surface expression of WNT5A in the indicated cell lines and primary cells was determined by flow cytometry using monoclonal antibody A-5 (Santa Cruz). **c, d**. The correlation between Ror2 mRNA expression and protein expression in cell lines was confirmed by Western blotting. ROR2 (antibody: Cell Signalling Technologies D3B6F) and actin bands in the indicated cell lines. **e.** ROR2 antibody (antibody: Santa Cruz H-1)) and GAPDH antibody bands in the indicated cell lines and primary cells. **f, g.** Separate channels and merge with bright field shown for WNT5A/ROR2 handover to AGS receiving cells by **f)** pCAF2 producing cells and **g)** AGS producing cells. **h**. The specificity of the Wnt5A/B antibody (antibody: ProteinTech 55184-1-AP) used in Fig. 1d was further examined by IF on pCAF2 cells stained with Phalloidin-FITC with an alternative anti-Wnt5A antibody (antibody: Santa Cruz A-5). Anti-mouse AF647 (Abcam) was used as a secondary antibody. The scale bar represents 10 μm.

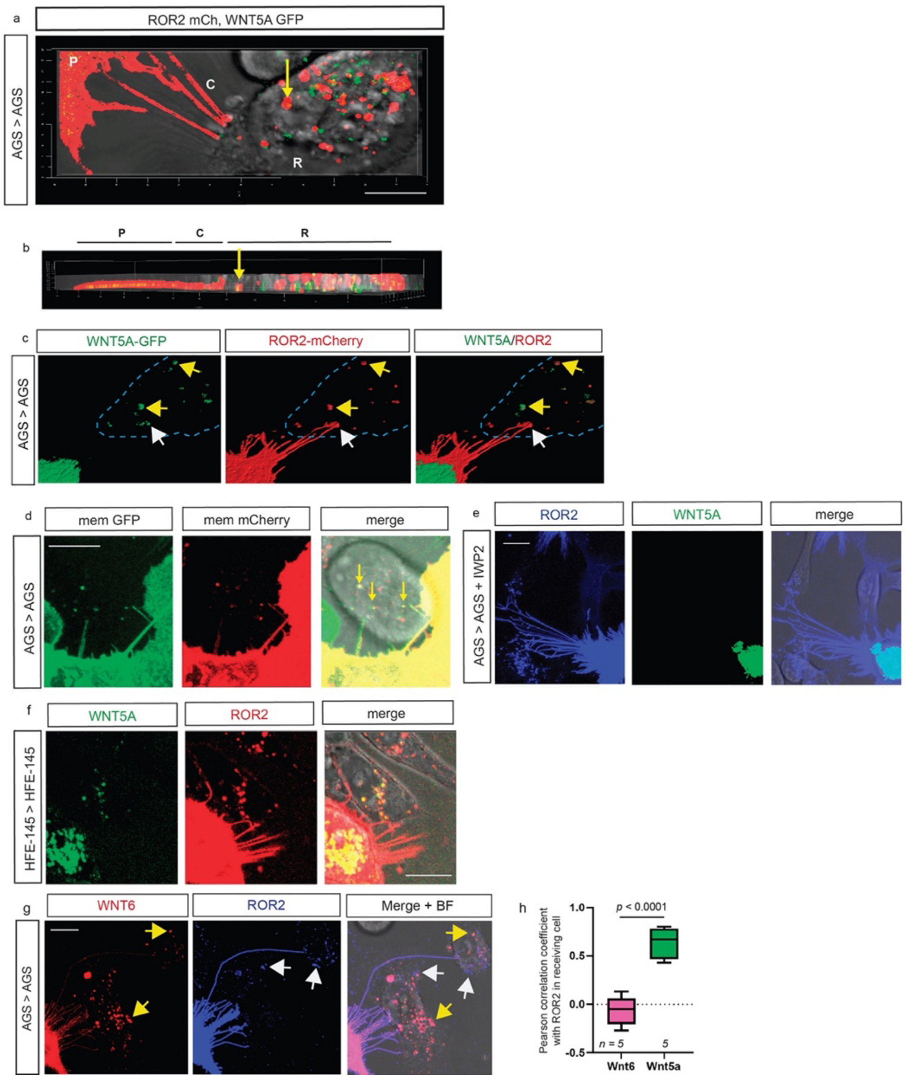

Figure S2. **a.** A producing AGS cell transiently transfected with ROR2-mCherry and WNT5A-GFP (P) is seen contacting a neighboring AGS receiving cell (R) identified in the BF image via cytonemes (C). The yellow arrow indicates a co-localizing ROR2/WNT5A complex in the receiving cell. The scale bar represents 10 µm. **b.** Orthogonal view of the image in a. The ROR2/WNT5A complex can be seen at the bottom of the receiving cell. **c.** AGS cell expresses ROR2-mCherry and WNT5A-GFP on cytoneme tips, which can be seen contacting a neighboring AGS receiving cell. 3D rendered version of the maximum projection from Fig. 2a. **d**. AGS cells were transiently transfected with membrane GFP and membrane mCherry as control fluorescent proteins and imaged using confocal microscopy. Membrane-bound vesicles co-localize in the receiving AGS cell, as indicated by the yellow arrows. The scale bar represents 10 µm. **e.** AGS cells were transiently transfected with ROR2 BFP and WNT5A GFP and cultured in the presence of 20µM of the porcupine inhibitor IWP2 for 24 hours. The scale bar represents 10 µm. **f.** HFE-145 gastric epithelial cells were transiently transfected with ROR2 BFP and WNT5A GFP and imaged by confocal microscopy. **g.** AGS cells transiently transfected with WNT6 mCherry and ROR2 BFP were co-cultured with untransfected AGS cells. **h.** Pearson correlation coefficient of either WNT6 or WNT5A co-localizing with ROR2 in a receiving cell calculated using Coloc2 in ImageJ. n = number of cells quantified. Significance was calculated using an unpaired t-test.

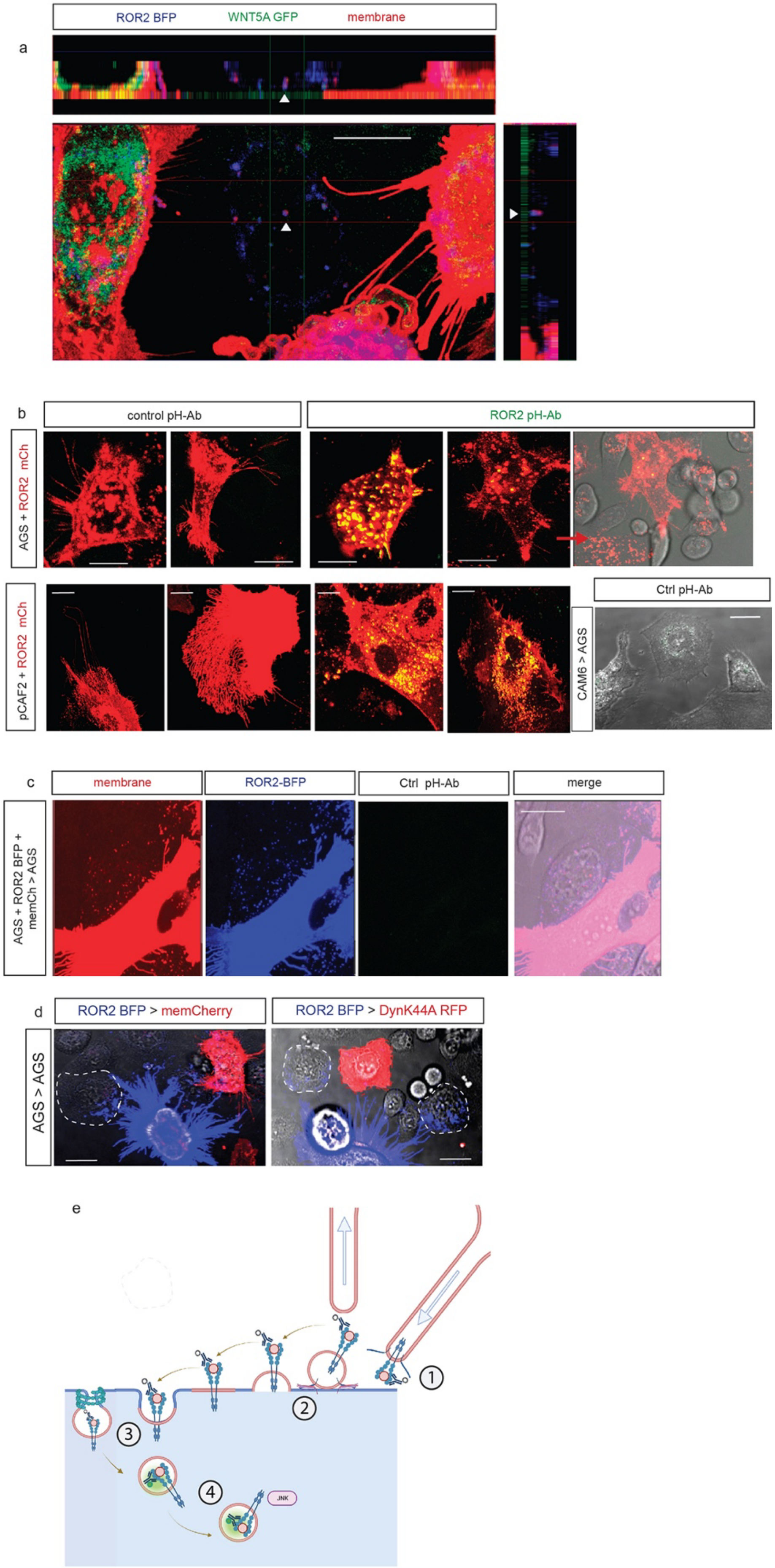

a
ROR2 BFP
WNT5A GFP
membrane
b
control pH-Ab
ROR2 pH-Ab
AGS + ROR2 mCh
pCAF2 + ROR2 mCh
Ctrl pH-Ab
CAM6 > AGS
c
membrane
ROR2-BFP
Ctrl pH-Ab
merge
AGS + ROR2 BFP + memCh > AGS
d
ROR2 BFP > memCherry
ROR2 BFP > DynK44A RFP
AGS > AGS
e
1
2
3
4
JNK

Figure S3:. **a.** Orthogonal view of the cells imaged in Fig. 3a. White arrowhead indicates the co-localization of the membrane, ROR2 BFP, and WNT5A GFP in the receiving cell. **b.** AGS cells or pCAF2 cells were transiently transfected with ROR2 mCherry and incubated with either control or anti-ROR2 antibody conjugated to pH-dependent green dye. Co-localization of endocytosed (but not membrane) ROR2 with antibody staining is seen in yellow. The expanded image on the right top row shows no ROR2-pH antibody binding to untransfected AGS cells. The image on the bottom right row shows the control pH-Ab on wild-type CAM6 and AGS co-culture. **c.** AGS cells transfected with membrane mCherry and ROR2-BFP were co-cultured with untransfected AGS cells, incubated in a control antibody labeled with a pH-dependent GFP dye for 20 hours, and live imaged by confocal microscopy. **d.** AGS cells were transiently transfected with ROR2-BFP and co-cultured with AGS cells expressing either membrane mCherry or RFP Dyn2^{K44A} for 24 hours. Cells were live imaged using confocal microscopy. **e.** Schematic showing the transfer of ROR2 from CAF source cells to AGS receiving cells vesicles. The pH-dependent ROR2 antibody binds to the extracellular CRD domain of ROR2, which is transferred from producing cell cytoneme to receiving cells associated with VAMP3. Dynamin2-dependent endocytosis follows, resulting in the ROR2 CRD domain facing into the lumen of the vesicle, as indicated by increased fluorescence of the pH-dependent antibody tag. This orientation places the ROR2 kinase domain in the cytoplasm of the producing and receiving cells.

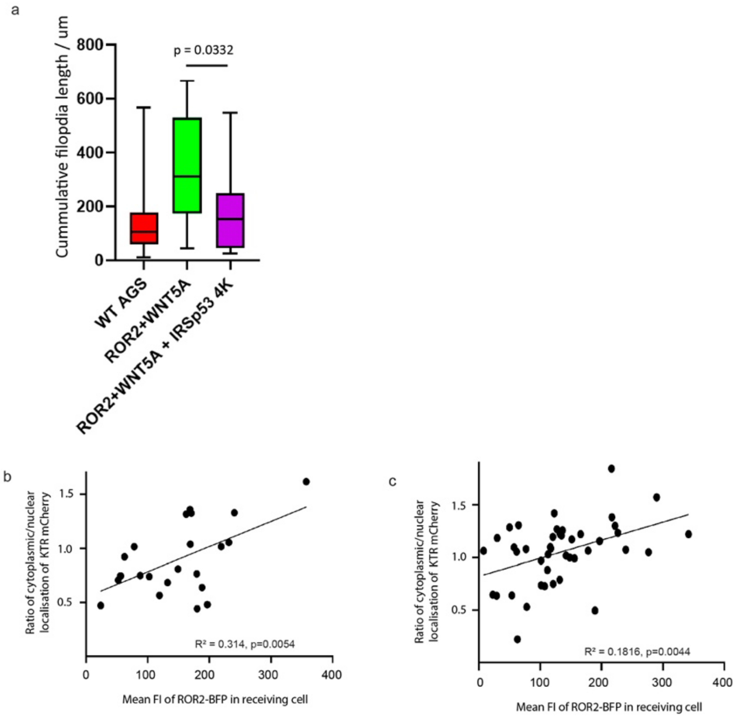

Figure S4. a. Cumulative filopodia length of producing AGS cells in Fig. 4e. AGS cells were transfected as indicated, and cumulative filopodia length was measured. Boxes represent 95% quartile, the centerline indicates the mean, and the whiskers indicate the range. n-values for each condition are as Fig. 4e. Significance was calculated using an unpaired t-test. **b.** The intensity of ROR2-BFP was quantified in AGS-B18 receiving cells following co-culture with pCAF2 transfected with ROR2-BFP (Fig. 4d). Values were plotted against corresponding cytoplasmic/nuclear JNK reporter localization, and a simple linear regression was performed. **c.** The intensity of ROR2-BFP was quantified in AGS-B18 receiving cells following co-culture with AGS transfected with ROR2-BFP (Fig. 4e). Values were plotted against corresponding cytoplasmic/nuclear JNK reporter localization, and a simple linear regression was performed.

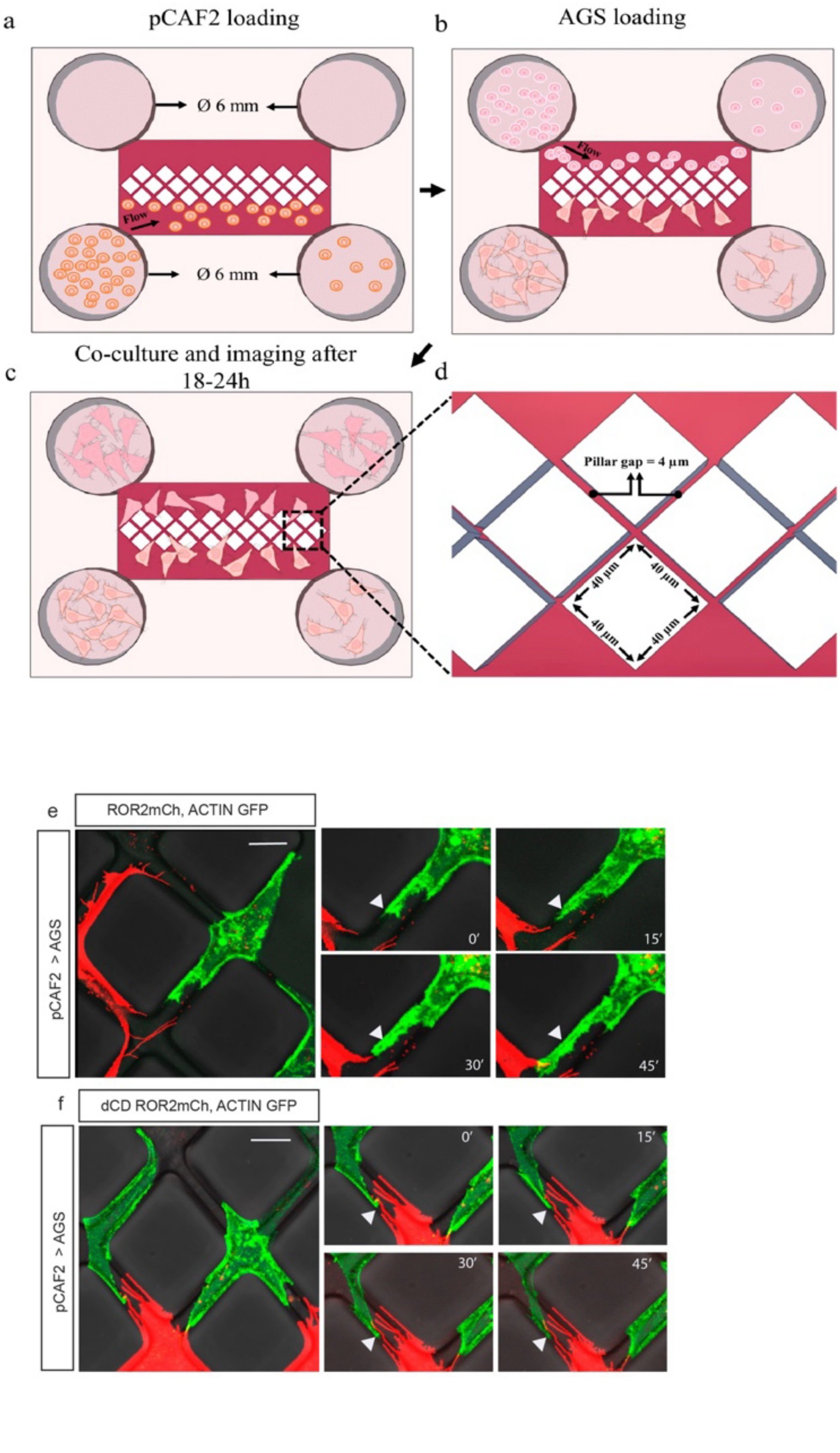
a
pCAF2 loading
Ø 6 mm
Flow
Ø 6 mm
b
AGS loading
Flow
c
Co-culture and imaging after 18-24h
d
Pillar gap = 4 µm
40 µm
40 µm
40 µm
40 µm
e
ROR2mCh, ACTIN GFP
pCAF2 > AGS
0′
15′
30′
45′
f
dCD ROR2mCh, ACTIN GFP
pCAF2 > AGS
0′
15′
30′
45′

Figure S5. Schematic of a bespoke microfluidic device enabling cell communication via filopodial networks, which was used to study pairwise cytonemal communication.
Microfluidic device fabrication was done using standard soft lithography techniques. Negative photoresist SU-8 3035 (MicroChem, Newton, MA) was spin-coated onto a silicon wafer at 4,500 rpm to a thickness of 30 µm. Pattern cross-linkage occurred via exposure to UV light through a chrome mask (Plate size x Thickness: 4"x4" x 0.09", ARC Quartz) (Micro Lithography Services Ltd). The microfluidic designs were drawn using AutoCAD 2021 (Autodesk). The device consists of four inlets for media/cell induction and a large chamber separated in half by a row of a diamond-shaped grid of pillars (40 µm x 40 µm). The pillars are spaced out with a 4 µm gap. Uncured polydimethylsiloxane (PDMS) (Sylgard 184) was poured onto the master (10:1 polymer to cross-linker mixture), degassed, and baked at 70 °C for 4 hours. The PDMS mold was then cut and peeled from the master. Four holes were punched using a 6 mm biopsy punch (Stiefel). The PDMS mold was then plasma bonded (Diener Zepto) to a thin cover slip (22 × 50 mm, 0.13 – 0.17 mm thick). Sterile PBS was immediately flushed through the device to increase the hydrophilic properties of PDMS. The device was placed under UV for 30 minutes before loading of samples.

a. On one side, pCAF2 cells are pipetted into large 6 mm wells from where they flow towards the pillar array. **b.** After 24h incubation, the AGS cells are introduced into the other side, allowing them to be placed at a close distance to the pCAF2 cells. **c.** After co-culture for 18-24 hours, cells are imaged at high-resolution. **d.** The planar microfluidic chamber is divided into two sides of equal volume by a linear array of diamond-shaped posts. The 4 µm pillar gaps are designed so that pCAF2 cells cannot migrate through the gaps, whereas filopodia can. **e** and **f.** pCAF2 cells transfected with either ROR2 mCherry or dCD ROR2 mCherry, respectively, were co-cultured with LifeAct-GFP expressing AGS cells in the microfluidic devices. Time-lapse images were taken using a confocal microscope, shown on the right-hand side, at indicated time points in minutes. The white arrow indicates the starting location of the extended AGS cell in each frame. The scale bar represents 10µm. Only a single image for each condition was collected.

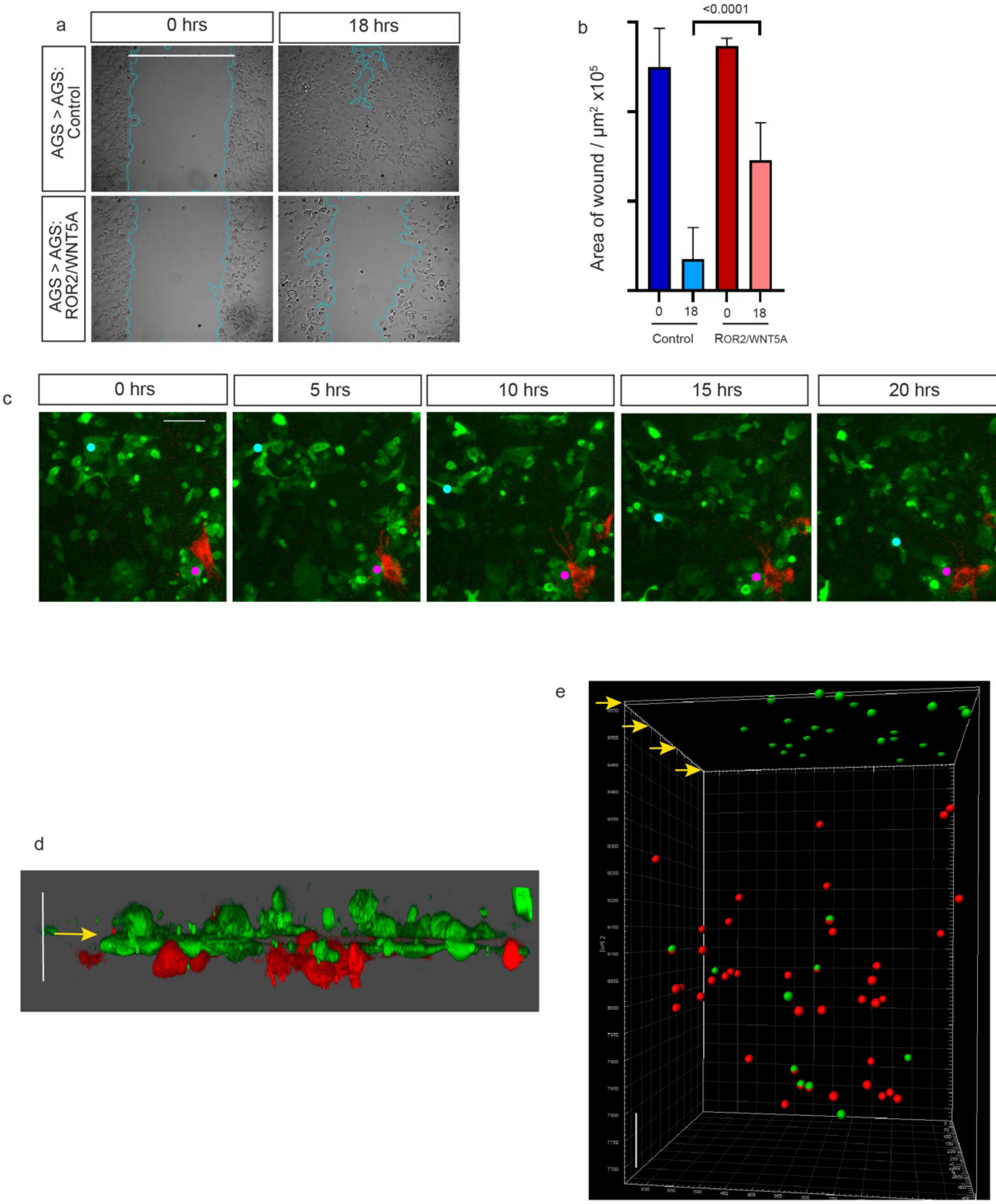
a
0 hrs
18 hrs
AGS > AGS: Control
AGS > AGS: ROR2/WNT5A
b
<0.0001
Area of wound / µm² x10⁵
0
18
0
18
Control
ROR2/WNT5A
c
0 hrs
5 hrs
10 hrs
15 hrs
20 hrs
d
e

Figure S6. ROR2/WNT5A overexpression induces a directional migration in AGS cells. **a.** Control AGS or AGS cells transfected with Ror2 and Wnt5a expression plasmids were cultured in Ibidi wound healing chambers for 24 hours prior to mitomycin C treatment and release. Dishes were imaged at release (0 hrs) and 18 hrs later. The scale bar represents 500μm. The area of the wound was calculated in ImageJ and is indicated by the blue line. **b.** The area of the wound was measured at the start and end of the experiment (18 hours), shown in Fig. S4a. Darker bar represents 0hrs, lighter bar represents 18 hrs. Blue bars represent control AGS cells, and red bars represent AGS cells transfected with ROR2 and WNT5A. **c**. Time lapses images taken at the indicated times of pCAF2 expressing ROR2 mCherry (red cells) co-cultured with AGS cells expressing membrane GFP. An individual AGS-receiving cell in close proximity to a pCAF2 cell is highlighted with a pink dot, whereas an individual AGS-receiving cell that starts at a distance to pCAF2 cells is highlighted with a light blue dot. The scale bar represents 100μm. **d.** AGS cells transiently transfected with membrane GFP are cultured in a monolayer in transwells with an 8μm pore size. pCAF2 cells transfected with membrane mCherry are cultured in 3D using Growdex at the bottom of the well, ensuring contact between the Growdex and the transwell. Cells were imaged 24 hours after co-culture. The yellow arrow indicates the position of the transwell membrane. mCherry-positive pCAF2 cells can be seen on the lower side of the transwell. GFP-positive AGS cells can be seen on both the top and lower sides of the transwell, indicating migration through the pores. The scale bar represents 100 μm. **e.** Representative image obtained from the invasion assay. AGS cells transiently transfected with membrane GFP are cultured in a monolayer in transwells with an 8μm pore size. pCAF2 cells transfected with either membrane mCherry, ROR2 mCherry, or dCD ROR2-mCherry are cultured in 3D using Growdex at the bottom of the well. Cells were imaged 72 hours after co-culture, and cells with a diameter in x, y, and z of more than 20μm were identified in Imaris. GFP-positive AGS cells were converted to dots, as shown, and the distance from the transwell (position indicated with yellow arrows) was calculated in Imaris.

Other supporting materials for this manuscript include the following:

Movie S1: The movie shows a rotation of a gCAM cell transfected with membrane GFP extending multiple filopodia in a 3D culture that contact AGS cells transfected with membrane mCherry, and transport multiple membrane-bound vesicles from producing to receiving cell (see also Fig. 1b and Fig. S1a).
